# Blue Light Potentiates Antibiotics in Bacteria via Parallel Pathways of Hydroxyl Radical Production and Enhanced Antibiotic Uptake

**DOI:** 10.1002/advs.202303731

**Published:** 2023-11-09

**Authors:** Leon G. Leanse, Carolina dos Anjos, Kylie Ryan Kaler, Jie Hui, Jeffrey M. Boyd, David C. Hooper, R. Rox Anderson, Tianhong Dai

**Affiliations:** ^1^ Wellman Center for Photomedicine Massachusetts General Hospital, Harvard Medical School Boston MA 02114 USA; ^2^ Health and Sports Sciences Hub University of Gibraltar, Europa Point Campus Gibraltar GX11 1AA Gibraltar; ^3^ Department of Biochemistry and Microbiology Rutgers University New Brunswick New Jersey 08901 USA; ^4^ Division of Infectious Diseases Massachusetts General Hospital, Harvard Medical School Boston MA 02114 USA

**Keywords:** antibiotics, antimicrobial resistance, blue light, synergy, wound infections

## Abstract

In the age of antimicrobial resistance, the urgency by which novel therapeutic approaches need to be introduced into the clinical pipeline has reached critical levels. Antimicrobial blue light (aBL), as an alternative approach, has demonstrated promise as a stand‐alone therapeutic method, albeit with a limited window of antimicrobial activity. Work by others indicates that treatment with antibiotics increases the production of reactive oxygen species (ROS) which may, in part, contribute to the bactericidal effects of antibiotics. These findings suggest that there may be potential for synergistic interactions with aBL, that similarly generates ROS. Therefore, in this study, the mechanism of aBL is investigated, and the potential for aBL to synergistically promote antibiotic activity is similarly evaluated. Furthermore, the translatability of using aBL and chloramphenicol in combination within a mouse model of *Acinetobacter baumanii* burn infection is assessed. It is concluded that porphyrins and hydroxyl radicals driven by “free iron” are paramount to the effectiveness of aBL; and aBL is effective at promoting multiple antibiotics in different multidrug‐resistant bacteria. Moreover, rROS up‐regulation, and promoted antibiotic uptake are observed during aBL+antibiotic exposure. Lastly, aBL combined with chloramphenicol appears to be both effective and safe for the treatment of *A. baumannii* burn infection. In conclusion, aBL may be a useful adjunct therapy to antibiotics to potentiate their action.

## Graphical abstract

The study demonstrates that porphyrins, free iron, and hydroxyl radicals drive antimicrobial blue light (aBL) efficacy. Exposure of aBL onto bacteria potentiated both the bactericidal and inhibitory effects of antibiotics because of ROS production, a ‘priming’ effect by aBL, and enhanced uptake. aBL also safely enhanced the efficacy of chloramphenicol in a mouse burn infection.

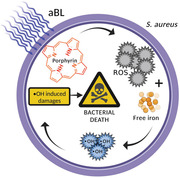

## 1 Introduction

In the age of antimicrobial resistance, it is now more important than ever to identify strategies that adequately control infectious diseases.^[^ ^1^, ^2^, ^3^ ^]^ Bacteria are particularly susceptible to becoming antibiotic resistant with numerous strains becoming multidrug‐resistant (MDR), indicating that they are resistant to a minimum of one within three antibiotic classes.^[^ ^4^ ^]^ Classically, bacteria are classified “resistant” if they are no longer susceptible to antibiotics at concentrations that are considered “safe” and thus clinically applicable.^[^ ^5^ ^]^ This is particularly important given that many antibiotics elicit significant host toxicity at elevated concentrations, and thus it is paramount that antibiotic concentrations administered do not exceed the defined therapeutic window.^[^ ^5^ ^]^

In recent years, our group has been involved with exploring non‐traditional methods for the treatment of infectious diseases. Specifically, we have been exploiting antimicrobial blue light (aBL), a “drug‐free” approach which has been demonstrated to be a potent microbicide, capable of eliminating a myriad of infectious agents both in vitro and in vivo.^[^ ^6^, ^7^, ^8^, ^9^ ^]^ It has the benefit of killing microbes safely, rapidly, and without the risk of resistance development.^[^ ^10^, ^11^ ^]^ However, like any light‐based antimicrobial method, an inescapable drawback of aBL relates to the longevity of its microbicidal effects, which are known to be short lived,^[^ ^6^, ^9^ ^]^ due to the short‐term lifespan of reactive oxygen species (ROS).^[^ ^12^ ^]^ The common hypothesis for how aBL elicits its microbicidal activity is via the excitation of endogenous porphyrins that induce reactive oxygen species (ROS) production.^[^ ^7^, ^13^, ^14^ ^]^ This in turn elicits damages within the microbial cell, leading to cell death.

While multiple studies have demonstrated ROS production (such as hydroxyl radicals [^•^OH]) in bacteria via blue light, validation of the mechanism has yet to be achieved). Studies have shown that some bactericidal antibiotics kill bacteria, in part, via ROS, with hydroxyl radicals being instrumental in inducing bacterial cell death.^[^ ^15^ ^]^ Specifically, antibiotics induce upregulation of the tri‐carboxylic acid cycle, to promote NADH production, which in turn boosts superoxide (^1^O_2_) production during oxidative phosphorylation. This “^1^O_2_ burst” can oxidize solvent accessible iron‐sulfur (Fe‐S) clusters resulting in cluster instability and degradation. This results in the release of ferrous iron ions. Non‐chelated ferrous iron is oxidized via a Fenton reaction, inducing hydroxyl radical (^•^OH) production that elicits bactericidal effects.^[^ ^15^ ^]^

Given that both antibiotics and aBL induce ROS production, we hypothesized that parallel pathways of bactericidal activity may exist to potentiate antibiotic activity in a similar manner as found previously.^[^ ^16^ ^]^ Therefore, the objectives of this study were to validate the mechanism of aBL, evaluate the potential for aBL to augment antibiotic activity in clinically important MDR bacteria in vitro, identify mechanistic insights relating to this augmentation, and validate the efficacy and safety of the aBL + antibiotic combination in vivo, and in a mouse model of burn infection model.

## 2 Results

### 2.1 Presence of Porphyrins in Bacteria and Hydroxyl Radical Production via Oxidation of “Free” Iron Mediate aBL Activity

**Figure 1:**
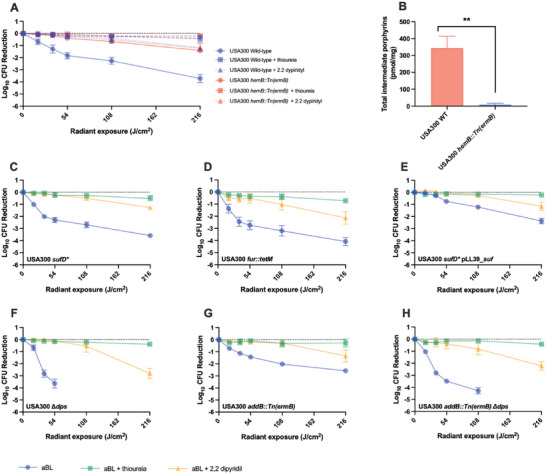
Killing kinetics of aBL with or with thiourea of pre‐treatment with 2,2‐dipyridil against A) USA300 Wild‐type and *hemB*:Tn (ermB). B) assessment of porphyrin quantification of the USA300 WT or USA300 hemB:Tn (ermB). Killing kinetics by aBL against C) USA300 SufD*, D) USA300 sufD* pLL39_suf, E) USA300 fur::tetM, F) USA300 dps, G) USA300 addB::Tn(ermB), and H) USA300 addB::Tn (ermB) dps. Statistical analysis: t‐test comparing aBL versus aBL+thiourea, aBL versus aBL+2,2‐dipyridil. Error bars: SEM.

In this study, for the first time, we identified the processes that drive aBL activity in bacteria, with the use of MRSA USA300 as a model organism. Initially, we sought to evaluate the role of porphyrins in the bactericidal activity of aBL, using a porphyrin deficient MRSA USA300 Δ*hemB* mutant (**Figure** **1**A,B). We found that the Δ*hemB* mutant was significantly less susceptible to the bactericidal effects of aBL with a 1.4‐log_10_ CFU reduction being reached after 216 J cm^−2^, compared with its parental wild‐type (WT) strain that achieved a 3.7‐log_10_ CFU reduction, with equivalent exposure (P<0.001).

We had detected both the production of H_2_O_2_ and •OH radicals following aBL illumination (Figure S1, Supporting Information). Therefore, we next evaluated the impact of •OH on the bactericidal effects of aBL. We found that co‐application of aBL with thiourea, a potent scavenger of •OH, severely compromised the efficacy of aBL, in both the WT and the Δ*hemB* mutant. For example, in the WT strain, suppression of •OH reduced the efficacy of aBL by > 1000‐fold, after 216 J cm^−2^ exposure, with only 0.4‐log_10_ CFU being reduced relative to 3.7‐log_10_ in the absence of thiourea (P < 0.001). Given the requirement of a Fenton reaction with iron to produce •OH radicals, we next assessed the consequence of chelating iron in MRSA USA300 using 2,2‐dipyridyl. Interestingly, we found there to be significant suppression in bactericidal effects elicited by aBL (> 100‐fold suppression), with only 1.23‐log_10_ CFU reduction compared with the 3.7‐log_10_ CFU reduction in the absence of 2,2‐dipyridyl, following 216 J cm^−2^ aBL exposure (Figure 1A). For the ΔhemB mutant, however, we did not observe any marked difference in efficacy in the presence of 2,2‐dipyridyl.

We next delved further into the mechanisms underpinning the aBL‐mediated Fenton reactions that were responsible for •OH production, and in consequence, bactericidal effects. Therefore, we sought to test our hypothesis that iron ions were supplied by Fe‐S clusters that were dissociated during aBL illumination. Using a Δ*sufD* mutant that was reduced in Fe‐S cluster production (USA300 sufD*), we evaluated bactericidal effects of aBL (with thiourea or 2,2‐dipyridyl) (Figure 1C). Surprisingly, we found the strain to be more susceptible to aBL than its parental WT strain (Figure 1A) and observed similar suppression of efficacy when ^•^OH radicals were suppressed, and iron was chelated. We were able to genetically complement this phenotype suggesting that the sufD* mutation was leading to the aBL sensitivity phenotype witnessed. was further reinforced when the sufD* strain was heterologously complemented with sufD which showed a suppression in aBL activity, likely because of less “free iron” (Figure 1D). Because the sufD* mutant contains more “free iron” than the parental WT strain, we hypothesized that this Fe pool may be targeted during aBL illumination (rather than iron leached from Fe‐S clusters).

The fur gene encodes for the ferric uptake regulator which represses transcription of Fe uptake genes in Fe replete conditions. The absence of Fur led to an increase in “free” Fe when compared to the WT strain (Figure S2, Supporting Information). We next tested the bactericidal effects of aBL on an Δ*fur* mutant (Δ*fur*::*tetM*). The Δ*fur* strain was more sensitive to aBL than the WT strain with 36 J cm^−2^ resulting in a 2.45‐log_10_ and 1.28‐log_10_ CFU reduction being achieved, respectively (Figure 1A,E). Dps is an Fe binding protein that also has a role in protecting DNA from Fenton catalyzed chemistry. We next evaluated the effectiveness of aBL on Δ*dps* mutant which has been shown to be deficient in protecting DNA from damage caused by Fenton chemistry.^[^ ^17^ ^]^ We found that a USA300 Δ*dps* mutant was highly susceptible to aBL, with a 3.63‐log_10_ CFU reduction being achieved after only 54 J cm^−2^ (Figure 1F), further reinforcing the importance of hydroxyl radicals in eliciting bactericidal effects. The Δ*dps* mutant did not appear to have an increase in non‐chelated iron (Figure S5, Supporting Information).

**Figure 2:**
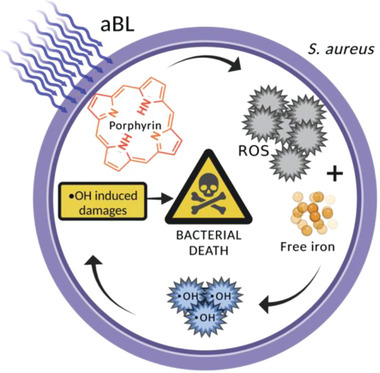
Diagrammatic representation of the proposed bactericidal mechanism of aBL against *S. aureus*.

Given the high susceptibility of the Δ*dps* mutant to aBL, we next tested aBL on an *addB*::Tn mutant that is defective in repairing DNA double‐strand breaks which can be caused by •OH radicals. Unlike with the Δdps mutant, we did not observe any improvement in bactericidal efficacy in the addB::Tn strain (Figure 1G). A Δdps addB::Tn double mutant displayed killing similar to that of the Δdps mutant (Figure 1H). The data presented in coordination with our previous findings resulted in a model wherein aBL treatment induces ROS that is photo‐induced via porphyrins that then oxidizes free iron (via Fenton chemistry) to generate ^•^OH radicals that induce the bactericidal effects (**Figure** **2**).

### 2.2 aBL Synergistically Enhanced Antibiotic Activity in Multidrug Resistant Bacteria

Data from others suggest that treatment with antibiotics may induce ROS production and the Fenton reaction, which may contribute to the bactericidal effects of antibiotics. The results presented demonstrate that treatment with aBL also induces ^•^OH production likely through Fenton chemistry. We hypothesized that these two parallel pathways of ROS induction would result in synergistic interactions. To test this hypothesis, we screened three different antibiotics with four, clinically important, and multidrug‐resistant bacterial species, *S. aureus, A. baumannii, P. aeruginosa*, and *E. coli*.

**Figure 3:**
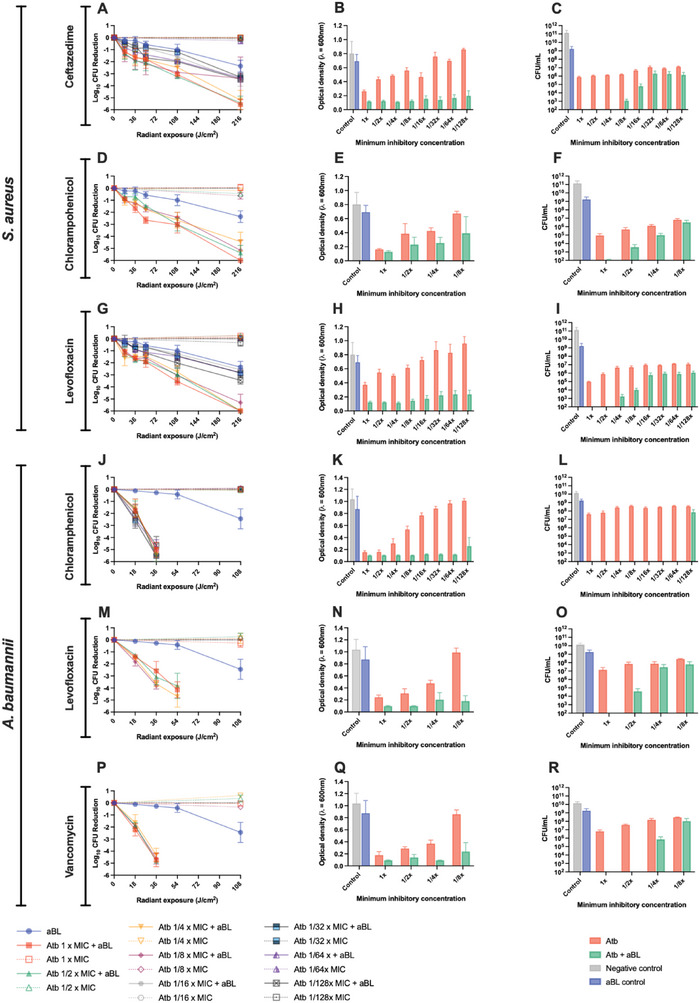
Killing kinetics of aBL + antibiotics against A,D,G) *S. aureus*, and (J,M,P) *A. baumannii*. Inhibition by aBL + antibiotics in (B,E,H) *S. aureus*, and (K,N,Q,I) *A. baumannii*, and post‐aBL bactericidal kinetics of antibiotics in (C,F,I) *S. aureus*, and (L,O,R) *A. baumannii*. For killing kinetics, a one‐way ANOVA (at 216 J cm^−2^) was performed with post‐hoc Bonferroni test comparing aBL versus aBL + antibiotics. For Inhibition studies, a one‐way ANOVA (at 216 J cm^−2^) was performed with post‐hoc Bonferroni test comparing antibiotics versus aBL + antibiotics. Error bars: SEM.

For *S. aureus*, we evaluated the dose‐response kinetics, inhibitory effects, and post‐aBL effects when aBL was combined with ceftazidime, chloramphenicol, or levofloxacin. For ceftazidime, we found that higher concentrations (approaching 1 x MIC) were associated with promoted killing (**Figure** **3**A). For example, at 1 x MIC and ½ x MIC in combination with 216 J cm^−2^, a > 5‐log_10_ CFU reduction was achieved, relative to aBL alone which only reduced the CFU by 2.4‐log_10_, although this was not found to be statistically significant (P ≥ 0.17). Ceftazidime alone, for the duration of aBL exposure (1 h) did not influence bacterial viability. While the killing kinetics of aBL did not appear to be significantly improved by ceftazidime, its effects on inhibition were highly significant and synergistic (Figure 3B). For example, we found that ceftazidime at 1/128 x MIC significantly and synergistically (S‐value 0.67; Figure S3A, Supporting Information) inhibited growth of S. aureus when aBL was co‐applied, with complete visible suppression and an Optical Density at 600 nm (OD_600_) of 0.19 compared with ceftazidime alone which has an OD_600_ of 0.86 (P = 0.006; data S6A, Supporting Information)). Both aBL alone and the untreated control did not affect growth significantly (P > 0.05). We next assessed the post‐aBL bactericidal effects (Figure 3C). We found that pre‐exposure to aBL + ceftazidime at 1 x MIC – 1/8 x MIC resulted in fewer viable bacteria relative to the treatment of bacteria with ceftazidime alone. For example, when aBL was combined with ceftazidime at ½ x MIC no viable bacteria were identified 24 h post‐treatment, compared with ceftazidime alone that has 1.2 × 10^6^ bacteria growing. While this demonstrated complete eradication of viable bacteria by ceftazidime when previously exposed to aBL, it was not found to be statistically significant (P = 0.52).

For chloramphenicol, the dose‐response kinetics were similarly assessed. As with ceftazidime, higher concentrations in combination with aBL, were associated with more efficient killing, relative to each therapy in isolation (Figure 3D). For example, at 216 J cm^−2^ exposure, aBL alone killed 2.36‐log_10_ CFU, relative to aBL + chloramphenicol (1 x MIC) that reduced the CFU by 6‐log_10_ (P = 0.0004). For inhibitory studies, we found that aBL synergistically promoted the inhibitory effects of chloramphenicol (Figure 3E). For example, at ½ x MIC the OD_600_ following aBL + chloramphenicol was 0.39 compared to chloramphenicol alone which was 0.67 (S‐value – 0.12; Figure S3B). With respect to post‐aBL bactericidal effects, we found them to be promoted relative to chloramphenicol alone (Figure 3F). For example, at 1 x MIC and ½ x MIC, there were fewer viable bacteria when co‐exposed with aBL, relative to chloramphenicol alone (6.6×10^1^ vs 9 × 10^5^ and 3.8×10^3^ vs 4.7 × 10^5^, respectively). As with the above antibiotics, for levofloxacin, the dose‐response kinetics were found to be improved in a dose‐dependent manner, with higher concentrations reflecting a more efficient killing response (Figure 3G). For example, at 1 x MIC, aBL + levofloxacin resulted in 6‐log_10_ CFU compared with aBL alone that resulted in a 2.36‐log_10_ CFU reduction (P = 0.0002). Enhanced inhibitory effects elicited by aBL were even more impressive, with concentrations of levofloxacin as low as 1/128xMIC resulting in significant (P = 0.002) and synergistic inhibition (S‐value 0.73; Figure S3C) of S. aureus with OD_600_ of 0.96 and 0.23 for the antibiotic alone and aBL + levofloxacin conditions, respectively (Figure 3H). Impressively, post‐aBL bactericidal effects were also observed, with concentrations of ½ x MIC levofloxacin + aBL resulting in no viable bacteria being identified compared with levofloxacin alone which had 8 × 10^6^ viable bacteria (Figure 3I). For all conditions, aBL alone and untreated controls did not influence the viability of S. aureus significantly (P > 0.05).

We next evaluated antibiotic enhancement of chloramphenicol, levofloxacin, and vancomycin by aBL in A. baumannii. For aBL + chloramphenicol, the dose‐response kinetics were significantly promoted relative to aBL alone (Figure 3J). For example, aBL + chloramphenicol (36 J cm^−2^; 1/64 x MIC) yielded a 4.8‐log_10_ CFU reduction, compared with aBL alone that reduced the viability by 0.41‐log_10_ CFU (P = 0.0004), demonstrating significant potentiation of antimicrobial activity. Additionally, inhibitory effects of chloramphenicol were highly improved by aBL expressing significant synergy (Figure 3K). For example, at 1/64 x MIC of chloramphenicol in combination with aBL at 36 J cm^−2^ an OD_600_ of 0.12 was observed relative to 0.97 following antibiotic alone (S‐value: 1.70; P = 0.007; Figure S3D, Supporting Information). With respect to post aBL bactericidal effects, we found that at at the concentration reflecting 1 x MIC – 1/64 x MIC concentration there was complete loss of viability in A. baumannii (Figure 3L).

For levofloxacin, we saw similar improvements to the dose‐response of aBL when levofloxacin was co‐administered. For example, at 36 J cm^−2 of^ aBL, combination with ¼ x MIC levofloxacin resulted in a 3.6‐log_10_ CFU reduction, compared with aBL alone which only reduced the viability by 0.41 log_10_ CFU (Figure 3M; P < 0.0001). Additionally, aBL significantly and synergistically improved the bacterial inhibition effect of levofloxacin (Figure 3N). For example, at 1/8 x MIC of levofloxacin in combination with 36 J cm^−2^ aBL, we found the OD_600_ to be 0.18, compared with levofloxacin alone which resulted in an OD_600_ of 0.99 (S‐value: 0.63; P = 0.01; Figure S3E, Supporting Information). With respect to the post‐aBL bactericidal effects, we found there to be some improvement when aBL was co‐administered. For example, at 1 x MIC and ½ x MIC of levofloxacin, we observed no detectable bacteria and 4 × 10^4^ bacteria, respectively, when combined with aBL at 36 J cm^−2^ (Figure 3O). When antibiotic alone was administered, however, there were 1.4 × 10^7^ and 7 × 10^7^ CFU of bacteria, respectively.

Last, for vancomycin in combination with aBL significantly improved the dose‐response relative to aBL alone (Figure 3P). For example, at 36 J cm^−2^ of aBL in combination with 1/8 x MIC of vancomycin, resulted in a 4.6‐log_10_ CFU reduction, relative to aBL alone, which only reduced the viability by 0.41‐log_10_ CFU (P < 0.0001). Additionally, the inhibitory effects of vancomycin were significantly and synergistically improved when combined with aBL (Figure 3Q). For example, vancomycin at 1/8 x MIC in combination with aBL at 36 J cm^−2^, an OD_600_ of 0.23 was observed, relative to vancomycin alone which yielded an OD_600_ of 0.86 (S‐value: 0.46; P = 0.06; Figure S3F, Supporting Information). For the post aBL‐bactericidal effects, we found improvements relative to antibiotic alone (Figure 3R). For example, at ½ x MIC of vancomycin in combination with 36 J cm^−2^ aBL, there were no viable bacteria identified, relative to vancomycin alone which had 3.7 × 10^7^ CFU of A. baumannii. In all studies described above (inhibitory and post‐aBL bactericidal studies), aBL alone did not have a significant influence on viability relative to the untreated control (P>0.05).

**Figure 4:**
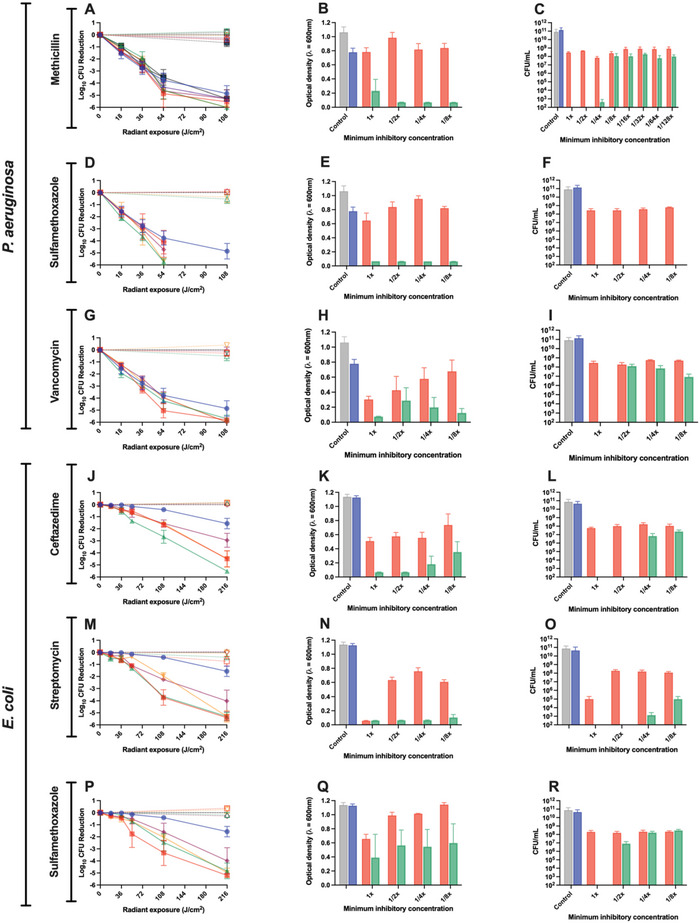
Killing kinetics of aBL + antibiotics against (A,D,G) *P. aeruginosa*, and (J,M,P) *E. coli*. Inhibition by aBL + antibiotics in (B,E,H) *P. aeruginosa*, and (K,N,Q) *E. coli*, and post‐aBL bactericidal kinetics of antibiotics in (C,F,I) *P. aeruginosa*, and (L,o,r)) *E.coli*. For killing kinetics, a one‐way ANOVA (at 216 J cm^−2^) was performed with post‐hoc Bonferroni test comparing aBL versus aBL + antibiotics. For Inhibition studies, a one‐way ANOVA (at 216 J cm^−2^) was performed with post‐hoc Bonferroni test comparing antibiotics versus aBL + antibiotics. Error bars: SEM.

Subsequently, we sought to evaluate the effects of aBL in combination with methicillin, sulfamethoxazole, and vancomycin against P. aeruginosa. Initially, we found that when aBL was combined with any of the 3 above mentioned antibiotics, the dose response of killing P. aeruginosa was not influenced, relative to aBL alone (**Figure** **4**A,D,G). The bacterial growth inhibition and post‐aBL bactericidal effects, however, were influenced significantly. For methicillin, we found synergistic enhancement of inhibition when combined with aBL (Figure 4B). For example, at 1/8xMIC in combination aBL at 36 J cm^−2^, the OD_600_ was 0.07 relative to methicillin alone which was 0.84 (S‐value: 0.51; P < 0.0001; Figure S3G, Supporting Information). The post‐aBL bactericidal effects of antibiotics were also improved (Figure 4C). For example, when ¼ x MIC of methicillin was combined with aBL at 36 J cm^−2^, only 4.3 × 10^2^ CFU of P. aeruginosa was detected, relative to methicillin alone which had 7 × 10^7^ (P < 0.001).

With respect to sulfamethoxazole, we also found growth inhibition and post‐aBL bactericidal effects to be improved (Figure 4E). For inhibition, combination of aBL at 36 J cm^−2^ with 1/8 x MIC of sulfamethoxazole, resulted in complete inhibition (0.06 OD_600_). In contrast, the use of sulfamethoxazole alone, which resulted in an OD_600_ of 0.82 (S‐value: 0.51; P < 0.001; Figure S3H, Supporting Information). For post aBL bactericidal studies, for example, we found that sulfamethoxazole in combination with aBL at 36 J cm^−2^, led to no detectable bacteria even at the concentration as low as 1/8^th^ MIC, while sulfamethoxazole alone yielded 6.3 × 10^8^ CFU of P. aeruginosa after treatment (P < 0.01; Figure 4F).

Lastly, when vancomycin was combined with aBL, we found bacterial growth inhibition and post aBL bactericidal effects to be improved (Figure 4H,I). For the inhibition studies, we observed improved inhibition by aBL + vancomycin relative to vancomycin alone (P = 0.02). For example, at 1/8 x MIC, vancomycin alone resulted in an OD_600_ 0.67. However, when combined with aBL at 36 J cm^−2^, the OD_600_ was 0.12 (S‐value: 0.5; Figure S3I, Supporting Information). The post‐aBL bactericidal effects were similarly affected. For example, at 1 x MIC, 2.8 × 10^8^ CFU was detected after treatment with vancomycin alone, compared with aBL + vancomycin which did not yield any detectable bacteria. In all experiments, aBL alone or untreated conditions did not significantly influence bacterial growth inhibition and post‐aBL bactericidal effects (P > 0.05).

Finally, we evaluated aBL in combination with ceftazidime, streptomycin, or sulfamethoxazole against E. coli. Unlike with P. aeruginosa, we did see improvements to the killing kinetics by aBL when in combination with any of the three antibiotics above. With ceftazidime, we found that 1/2xMIC in combination with aBL at 216 J cm^−2^ resulted in a 5.5‐log_10_ CFU reduction in E. coli, compared with aBL alone which resulted in only a 1.6 log_10_ CFU reduction (P < 0.0001; Figure 4J). In the inhibition studies, there was significant and synergistic inhibition observed with aBL + ceftazidime (Figure 4K). For example, when 1/4xMIC of ceftazidime was combined with aBL at 216 J cm^−2^, it resulted in an OD_600_ of 0.18, compared with ceftazidime alone which resulted in an OD_600_ of 0.55 (S‐value: 0.32; P = 0.03; Figure S3J, Supporting Information). The post‐aBL effects were also promoted. For example, at 1/2xMIC of ceftazidime, there were no viable bacteria identified when combined with aBL, compared with ceftazidime alone which had 1 × 10^8^ CFU of bacteria remaining (Figure 4L).

For the streptomycin studies, we similarly observed improvements in the aBL dose‐response when combined with streptomycin (Figure 4M). For example, when 1/8xMIC of streptomycin was combined with 216 J cm^−2^ aBL, with a 4‐log_10_ CFU reduction was found, compared to aBL alone which only reduced the bacterial viability by 1.6‐log_10_ CFU (P = 0.05). Bacterial inhibition studies were similarly positive. For example, when 1/8xMIC of streptomycin was combined with aBL at 216 J cm^−2^, there was an OD_600_ of 0.1 compared with 0.6 that was achieved by antibiotic alone (S‐value: 0.44; P < 0.0001; (Figure 4N; Figure S3K, Supporting Information). The post‐aBL bactericidal effects were also improved. For example, at 1 x MIC and ½ x MIC of streptomycin in combination with aBL 216 J cm^−2^, there were no viable bacteria detected, relative to streptomycin alone which had 1 × 10^5^ and 1.9 × 10^8^ CFU remaining, respectively (Figure 4O).

We then studied the effects of sulfamethoxazole in combination with aBL. Significant enhancement in bacterial killing by aBL + sulfamethoxazole was observed. For example, when sulfamethoxazole at 1xMIC combined with 216 J cm^−2^ aBL, there was a 5.2‐log_10_ CFU reduction in bacteria, compared with aBL alone that reduced the bacterial CFU by 1.6 log_10_ (P = 0.0001; Figure 4P). For the inhibition studies, there was synergistic enhancement of growth suppression when aBL was applied with sulfamethoxazole Figure 4Q). For example, at 1/8xMIC of sulfamethoxazole combined with aBL at 216 J cm^−2^, the OD_600_ was 0.6 compared with sulfamethoxazole alone which yielded an OD_600_ of 1.14 (S‐value: 0.47; P = 0.1; Figure S3L, Supporting Information). For the post‐aBL bactericidal effects, there was some improvement by aBL + sulfamethoxazole, relative to antibiotic alone. For example, at 1xMIC sulfamethoxazole combined with 216 J cm^−2^ aBL, no viable bacteria were identified, however, with antibiotic alone there were 2 × 10^8^ CFU of bacteria identified. As with above, aBL alone did not influence bacterial growth or post aBL bactericidal effects, relative to the untreated control (P > 0.05; Figure 4R).

### 2.3 Blue Light Primed Bacteria to Promote Bactericidal and Inhibitory Effects by Antibiotics

**Figure 5:**
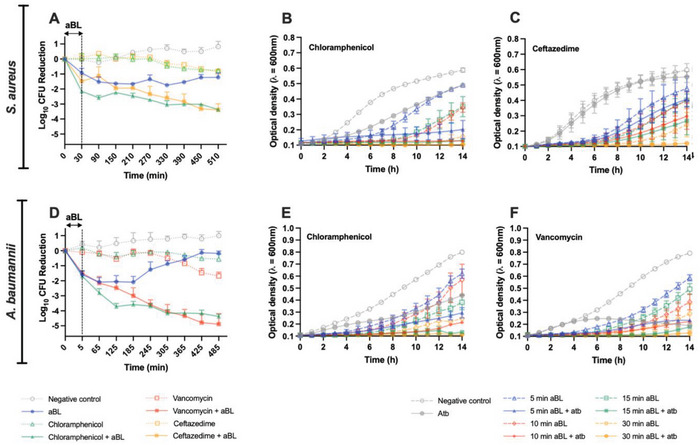
Bactericidal kinetics of antibiotics post‐aBL illumination over 8 h (510 min) for A) *S. aureus*, and D) *A. baumannii*. Inhibitory kinetics of B) chloramphenicol and C) ceftazidime in *S. aureus* post aBL illumination. Inhibitory kinetics of E) chloramphenicol, and F) vancomycin in *A. baumannii*. Statistical analyses: one‐way ANOVA with post‐hoc Bonferroni test comparing antibiotics versus aBL + antibiotics. Error bars: SEM.

In this study, we sought to test our hypothesis that co‐administration of aBL primes bactericidal and inhibitory effects of antibiotics irrespective of antibiotic or its bactericidal/bacteriostatic classification (**Figure** **5**A–F). Therefore, using *S. aureus* and *A. baumannii*, as representative Gram‐positive and Gram‐negative bacteria, respectively, together with a bacteriostatic antibiotic (chloramphenicol) and 2 bactericidal antibiotics (ceftazidime and vancomycin) that are not typically indicated for each bacterial type (e.g., vancomycin for *A. baumannii*, and ceftazidime for *S. aureus*); we assessed how exposure to aBL influences subsequent killing and inhibition of these bacteria.

For bactericidal studies, we found that for both bacteria (and antibiotics) exposure to aBL stimulated subsequent bactericidal effects that were significantly greater than those seen by antibiotic alone. For *S. aureus*, exposure to 108 J cm^−2^ aBL (30 min) in combination with ceftazidime at 1xMIC resulted in a reduction of 1.5‐log_10_ CFU. However, as bacteria continued to be incubated with ceftazidime (after completing aBL exposure), bactericidal effects continued to be observed (Figure 5A). For example, following 8 h of incubation with ceftazidime, a total of 3.4‐log_10_ CFU were killed, demonstrating an increase in bacterial killing by 1.9‐log_10_ CFU. With respect to ceftazidime alone, however, 8 h of incubation only reduced 0.8‐log_10_ CFU (*P* < 0.001). Impressively, with aBL alone, 0.9 log_10_ CFU reduction was observed following an exposure of 108 J cm^−2^, and CFU reduction continued to increase to 1.5‐log_10_ at 60 min after aBL exposure, suggesting the presence of post‐aBL bactericidal effects immediately following illumination. However, at the completion of the 8 h incubation, there was some bacterial regrowth, totaling the viability loss to only 1.2‐log_10_ CFU. Results from the chloramphenicol studies were similar. Exposure to 108 J cm^−2^ (30 min) aBL reduced the bacterial viability by 2.14 log_10_ CFU, and bacterial viability reduction increased to 3.4 log_10_ CFU following the 8 h incubation, which was significantly greater compared to chloramphenicol alone which only reduced the bacterial viability by 0.76 log_10_ CFU.

For *A. baumannii*, the observed results were like those found in *S. aureus*. When 18 J cm^−2^ aBL (5 min) was combined with vancomycin at 1×MIC, a 3.4‐log_10_ CFU reduction was achieved immediately after aBL exposure was discontinued (initial viability loss of 1.5‐log_10_ CFU; total viability loss of 4.9‐log_10_ CFU), demonstrating a significant priming of subsequent vancomycin activity, compared with vancomycin alone which only reduced the total viability over 8 h by 1.7‐log_10_ (*P* = <0.0001; Figure 5D). For aBL + chloramphenicol, similar findings were observed. Just two hours following the completion of aBL exposure, the viability of *A. baumannii* was reduced by 1.9‐log_10_ CFU (total loss of 3.7‐log_10_ CFU). At the end of the 8 h experiment, the total viability loss of *A. baumannii* was 4.4‐log_10_ CFU. When treated with chloramphenicol alone, however, the total viability loss was only 0.6‐log_10_ CFU. Unsurprisingly, with aBL alone, following an initial decrease in viability, bacteria subsequently “regrew” almost completely following 7 h incubation post aBL, demonstrating only a 0.21‐log_10_ CFU reduction by the end of the 8 h experiment.

We next assessed the how aBL dosimetry affected antibiotic‐mediated inhibition of bacterial growth. For *S. aureus*, we found that higher aBL exposures were associated with greater inhibition, irrespective of antibiotic concentrations. However, at lower aBL exposures, we could observe “priming” effects more clearly, whereby aBL positively affected antibiotic‐mediated inhibition, without compromising inhibition on its own. For example, following 18 J cm^−2^ aBL exposure (5 min), the bacterial growth was significantly delayed compared with either aBL or chloramphenicol alone (*P* = 0.0001; Figure 5B). After 14 h incubation (post aBL illumination), the OD_600_ of bacterial suspension was 0.2, in comparison to the bacteria exposed to either aBL or antibiotic alone both grew to an OD_600_ of 0.47 and 0.55, respectively. Meanwhile, the OD_600_ of untreated bacteria grew to 0.59. For aBL + ceftazidime, findings reflected those found with aBL + chloramphenicol. However, higher aBL exposures were required to observe the “priming” effect that was similarly found in aBL + chloramphenicol above. For example, following 108 J cm^−2^ of exposure (30 min), an OD_600_ of 0.27 was observed with aBL + ceftazidime, compared with aBL alone and ceftazidime alone which had an OD_600_ of 0.4 and 0.552, respectively (*P* <0.001; Figure 5C). In parallel, the untreated control had an OD_600_ of 0.6.

Similarly, we observed “priming” inhibitory effects of antibiotic in *A baumannii* when pre‐exposed to aBL. For aBL + chloramphenicol, for example, at just 18 J cm^−2^ of illumination (5 min), we saw significant inhibition by chloramphenicol relative to antibiotic or aBL alone, with an OD_600_ of 0.29, 0.43, and 0.62, respectively being achieved (*P* = 0.0005; Figure 5E). For aBL + vancomycin, the influence of aBL on the priming effects was also evident, however, it appeared to be less dramatic than what was seen with aBL + chloramphenicol. For example, at 18 J cm^−2^ of aBL (15 min) in combination with vancomycin at 1×MIC, following 10 h of incubation, there was an OD_600_ of 0.12 in bacterial suspensions compared with antibiotic alone which led to an OD_600_ of 0.22, and aBL alone which was 0.26 (*P* < 0.0001; Figure 5F). The untreated control had an OD_600_ of 0.6 following 10 h of incubation.

### 2.4 Co‐Administration of aBL and Antibiotics Increased ROS Production

**Figure 6:**
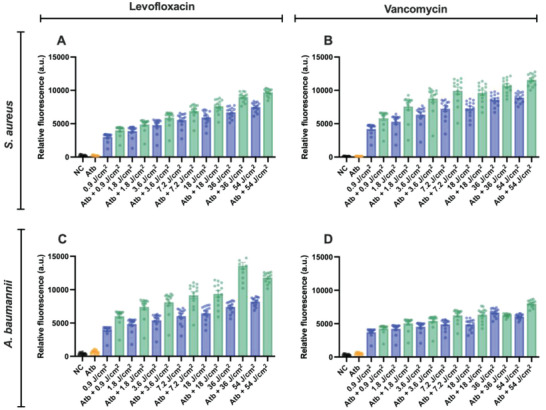
Assessment of generic ROS production by 2′, 7′‐dichlorofluorescein diacetate (DCFH‐DA) following different aBL exposures (0.9 J cm^−2^ – 54 J cm^−2^) in combination with different antibiotics. A) *S. aureus* with aBL, aBL + levofloxacin, levofloxacin alone, or negative control. B) *S. aureus* with aBL, aBL + vancomycin, vancomycin alone, or negative control. C) *A. baumannii* with aBL, aBL + levofloxacin, levofloxacin alone, or negative control. B) *A. baumannii* with aBL, aBL + vancomycin, vancomycin alone, or negative control. Statistical analyses: one‐way ANOVA with post hoc Bonferroni correction comparing aBL versus aBL + antibiotics. Error bars: SEM.

We wanted to determine whether there was any significant increase in generic ROS when aBL was co‐administered with antibiotics. For this study we used *S. aureus* and *A. baumannii* as representative bacteria and two representative bactericidal antibiotics: levofloxacin and vancomycin. In *S. aureus*, we found that, when either levofloxacin or vancomycin was combined with aBL, there was significant increases in ROS production relative to the sum of ROS production by aBL and antibiotic alone. Impressively, aBL generated ROS production at exposures as low as 0.9 J cm^−2^ (e.g., 4235 relative fluorescence units [RFU], compared with untreated which was 142 RFU (**Figure** **6**A,B). Higher exposures were associated with higher RFUs indicative with higher levels of ROS production. When aBL at 54 J cm^−2^ was combined with vancomycin at 1×MIC, for example, the ROS production reached 11 592 RFU compared with aBL alone which was 8936 RFU, indicating an increase of 23% (*P* < 0.001). ROS production by antibiotic alone at 1 x MIC was only 166 RFU, which was not significantly different from the untreated control (142 RFU). Results obtained from aBL + levofloxacin were highly similar to those found in the above aBL + vancomycin.

For *A. baumannii*, similar results were obtained to those found in *S. aureus* (Figure 6C,D). For example, when 54 J cm^−2^ of aBL was applied with vancomycin at 1×MIC, an 8002 RFU was achieved, relative to aBL alone which yielded an RFU of 6065, indicating a 24.3% increase in ROS production (*P* < 0.001). ROS produced by vancomycin alone was found to be significantly upregulated relative to the untreated control with RFUs of 594 and 471, respectively, indicating a 20% increase in ROS production (*P* = 0.0009). With respect to aBL + levofloxacin results, they were found to be like those achieved with aBL + vancomycin.

### 2.5 Porphyrins are Important for “Priming” Bactericidal Effects of Antibiotics in Bacteria by aBL

**Figure 7:**
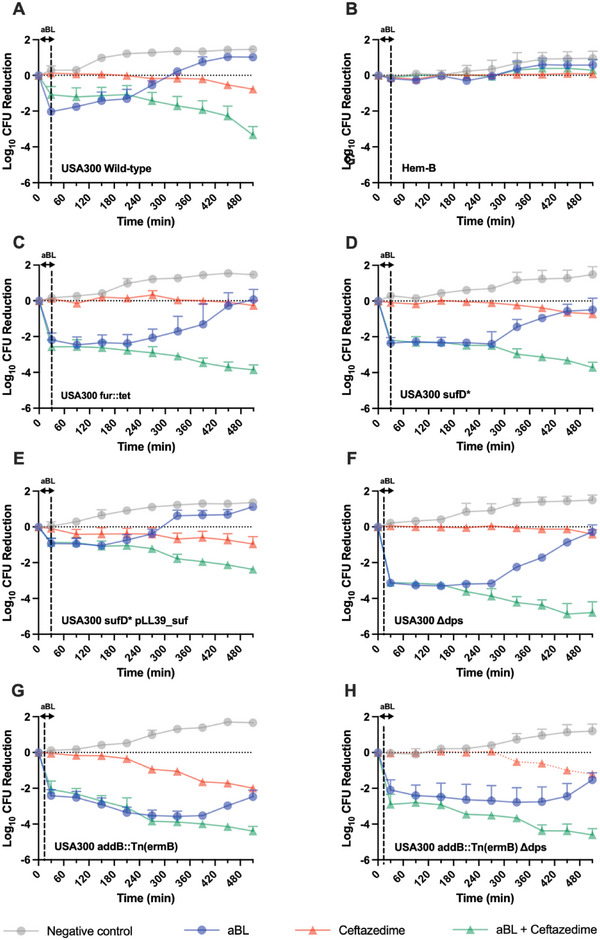
Bactericidal kinetics of ceftazidime following aBL (108 J cm^−2^) in A) USA300 wild‐type, B) hemB mutant, C) USA300 fur::tet, D) USA300 sufD*, E) USA300 sufD* pLL39_suf, F) USA300 dps, G) USA300 addB::Tn(ermB), H) USA300 addB::Tn(ermB)dps. Statistical analyses: one‐way ANOVA with post‐hoc Bonferroni test comparing ceftazidime versus aBL + ceftazidime.

Using *S. aureus* as the model species, in this study, we determined the influence of porphyrins, defective Fe‐S cluster synthesis, and altered cytosolic iron loads on priming bactericidal effects of antibiotics by aBL. Of all the mutants tested (**Figure** **7**A–H), the hemB mutant was the only one that was incapable of showing any priming effects when treated with aBL + ceftazidime (Figure 7B). For example, shining aBL for 108 J cm^−2^ (30 min) did not elicit any highly significant bactericidal effects, with the viability only being reduced by 0.27‐log_10_ CFU, and with no subsequent bactericidal effects being evident. Interestingly, we did not observe any reduction in viability with ceftazidime alone over the course of 8 h of incubation. When aBL was combined with ceftazidime, we similarly did not see any reduction in viability whatsoever; there was, in fact, a slight increase in the numbers of viable bacteria (by 0.29‐log_10_ CFU). In the untreated control, bacterial viability increased by 0.95‐log_10_ CFU.

With respect to the Δ*fur* strain, we observed significant reduction in bacterial CFU (> 2‐log_10_) with both aBL alone and aBL + ceftazidime (Figure 7C). However, when treated with aBL alone, bacteria completely “re‐grew” at the end of the 8 h incubation. When aBL was combined with ceftazidime, but after 8 h there was a total reduction of 3.86‐log_10_ CFU (*P* = 0.02), which decreased the viability by 1.29‐log_10_ CFU after aBL exposure ceased, thus demonstrating a priming effect.

For the Δ*sufD* mutant, we found that aBL alone and aBL + ceftazidime reduced the CFU by 2.4‐log_10_, after an exposure of 108 J cm^−2^ (30 min; Figure 7D). Following 8 h of incubation, the aBL‐treated bacteria regrew completely, whereas in the case of aBL + ceftazidime, there was a 3.7‐log_10_ CFU reduction, which increased from 1.36 after aBL exposure ceased.

Similar trends were seen with the Δ*dps* mutant, which was highly susceptible to aBL and aBL + ceftazidime, with a total killing of > 3‐log_10_ CFU following an exposure of 108 J cm^−2^ (30 min illumination; Figure 7F). Interestingly, as with the other “susceptible” mutants, aBL alone allowed a complete regrowth compared with aBL + ceftazidime, which reduced the viability by 4.8‐log_10_ CFU which was 1.68‐log_10_ CFU lower in viability than immediately following aBL exposure. With respect to the WT strain (Figure 7A), the results were very similar to the mutant results (except for the Δ*hemB* mutant) with aBL‐treated sample completely re‐growing after 8 h incubation, compared with the aBL + ceftazidime group that reduced the viability by 3.3‐log_10_ CFU, following an 8 h incubation, which further reduced the viable bacteria by 2.25‐log_10_ CFU after aBL exposure stopped. These findings thus suggest that the priming effect of aBL with antibiotics is contingent on the presence of porphyrins.

### 2.6 Blue Light Promoted Accumulation of Vancomycin Into the Cell Wall of Bacteria

**Figure 8:**
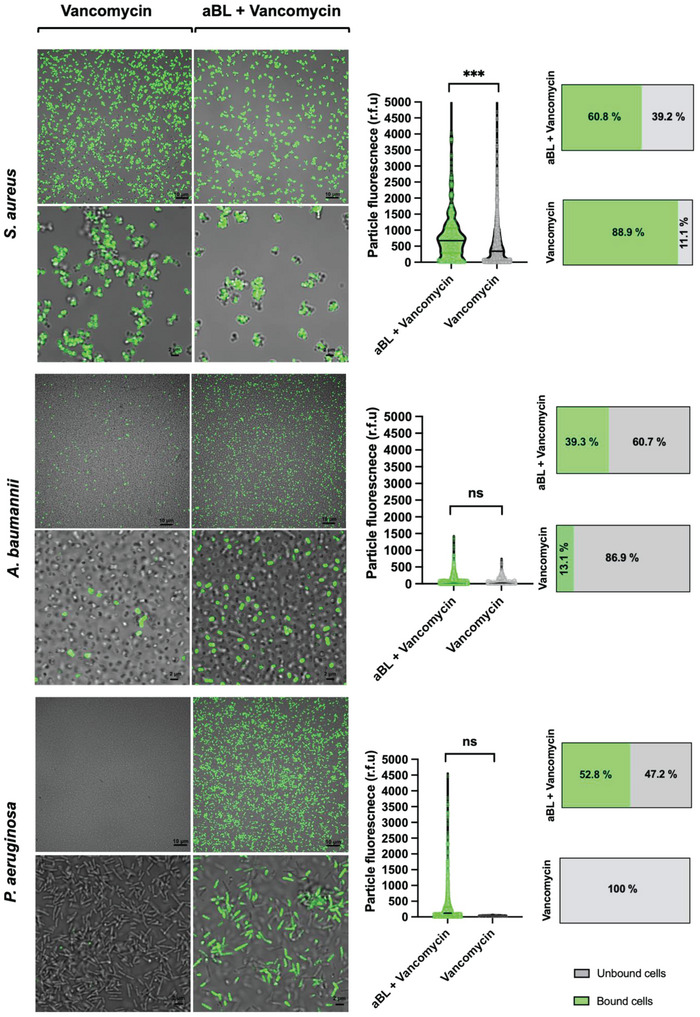
Confocal images of *S. aureus, A. baumannii*, and *P. aeruginosa*, following exposure to the fluorescent vancomycin‐bodipy conjugate in the presence or absence of aBL. For each species, there include quantification of the sub‐population of bacteria that are fluorescence, as well as violin scatter plots illustrating mean particle fluorescence. Statistical analyses: unpaired t‐test comparing vancomycin alone versus aBL + vancomycin.

In this study, we determined whether the observed priming effect by aBL to potentiate action of antibiotics may be due, at least in part, to promoted uptake of antibiotics. Specifically, we observed that antibiotics that are exclusively used against Gram‐positive bacteria (e.g., vancomycin and methicillin), due to a lack of an outer membrane, could indeed function against Gram‐negative bacteria, when aBL was exposed. Those antibiotics are not used against Gram‐negative bacteria, due to their robust outer membrane which limits antibiotic uptake. Therefore, we hypothesized that outer membrane permeabilization by aBL may promote antibiotic accumulation. We used *S. aureus, A. baumannii*, and *P. aeruginosa* as a model to determine the role of aBL in facilitating uptake of vancomycin into the cell wall of bacteria (**Figure 8**; see below). Initially, we found that NPN fluorescence intensified with increasing aBL exposures in *E. coli, A. baumannii, and P. aeruginosa*, suggesting that aBL induced outer membrane permeabilization, which would suggest that aBL may facilitate uptake of antibiotics (Figure S4, Supporting Information).

As evident in Figure 8, for For *S. aureus*, exposure to aBL reduced the population of bacteria that accumulated vancomycin within their cell wall. However, we also found that, at a single cell level, there was an average fluorescence of 672 RFU in *S. aureus* that was previously exposed to aBL and 342.3 RFU in *S. aureus* without aBL (*P* < 0.001). For *A. baumannii* and *P. aeruginosa*, however, we found that pre‐exposure to aBL significantly increased the population of bacteria that accumulated vancomycin in their cell walls relative to vancomycin alone. However, there was no difference in the amount of vancomycin, at a single cell level, that entered cells.

For *A. baumannii*, only 13% of the population accumulated vancomycin when in the absence of aBL (Figure 8). However, post‐aBL exposure, the population increased to 39%, demonstrating a significant increase in numbers of bacteria that took up vancomycin. For *P. aeruginosa*, in the absence of aBL, there was no apparent uptake of vancomycin into cells. Remarkably, when vancomycin was applied after aBL exposure, the uptake increased dramatically with 52.8% of bacteria having taken up the molecule. All the findings thus suggest that improved uptake of antibiotics mediated by aBL might play a role in facilitating the “priming” effect that was seen when aBL was exposed concomitant with antibiotics.

### 2.7 Combined aBL and Chloramphenicol Safely and Effectively Treated an A. *Baumannii* Burn Infection in a Mouse Model

**Figure 9:**
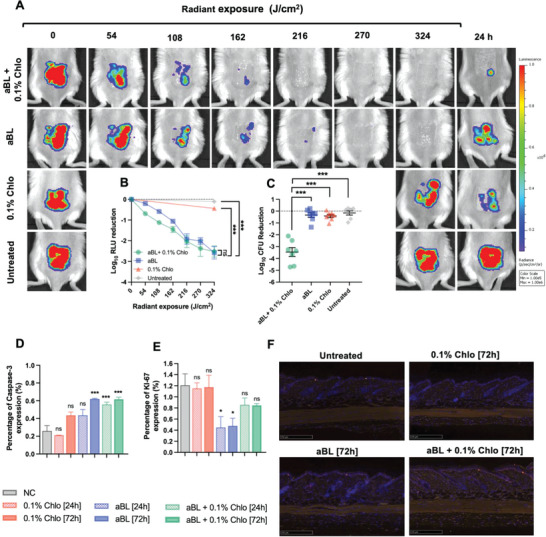
In vivo *A. baumannii* mouse burn infection study showing A) representative bioluminescent images of mice treated with aBL + 0.1% chloramphenicol, aBL, 0.1% chloramphenicol, and untreated (PBS vehicle). B) Quantification of the RLU following different aBL aliquots (54 −324 J cm^−2^). C) CFU analyses 24 h after treatment, with each data point indicating a different animal. D) Quantification of percentage caspase‐3 positive cells post aBL or aBL + chloramphenicol, chloramphenicol alone, or untreated. E) Quantification of percentage of Ki‐67 positive cells. following aBL, aBL+ chloramphenicol, chloramphenicol alone, or untreated. F) Representative images of Caspase‐3 assessments in naïve mouse skin. Statistical analyses: one‐way ANOVA with post‐hoc Bonferroni test comparing aBL versus aBL + chloramphenicol or chloramphenicol versus aBL + chloramphenicol. *P<0.05, ***P<0.001.

In this study, we tested our hypothesis that aBL combined with antibiotics can effectively improve the treatment of infections. We combined aBL with chloramphenicol to treat *A. baumannii* burn infections in a mouse model. We selected chloramphenicol as it demonstrated significant synergy with aBL against *A. baumannii*, and it has been used previously to treat infections topically.^[^ ^18^ ^]^ Initially, we assessed the efficacy, in real‐time, of aBL, chloramphenicol, and its combination in treating burn infection caused by *A. baumannii*, using bioluminescence imaging (**Figure** **9**A,B). For the treatment with aBL alone, we found that after the maximum aBL exposure (324 J cm^−2^) a 2.5‐log_10_ reduction in bioluminescence signal [(relative luminescence units (RLU)] in mice was achieved. Combining aBL with chloramphenicol, however, did not improve the killing in real‐time, with an equivalent loss of *A. baumannii* viability being achieved. Chloramphenicol alone reduced the viability by 0.42‐log_10_ RLU, and the untreated control lost 0.1‐log_10_ RLU during the experiment.

Impressively, however, having assessed the bacterial burn in mice 24 h following treatment, we found a 3.7‐log_10_ CFU reduction in the group treated with both aBL + chloramphenicol, relative to the untreated control (Figure 9C). The infection in the aBL alone group, however, almost completely reverted, illustrating only a 0.25‐log_10_ CFU reduction, and chloramphenicol alone only reduced the CFU by 0.35‐log_10_. Thus, aBL + chloramphenicol was found to be synergistic (S‐value: 0.29; *P* < 0.001).

We next assessed safety of aBL + chloramphenicol, applying the doses we used during the treatment experiments. We initially evaluated cellular apoptosis in naïve mouse skin after applying aBL alone and aBL + chloramphenicol (Figure 9D,F). For aBL alone, we found a marginal increase (0.17% increase; *P* > 0.05) in apoptotic cells relative to the untreated control. At 72 h, however, there were slightly higher number of apoptotic cells (0.62%; or 0.36% increase) relative to the untreated control. For chloramphenicol alone, at 24 h post exposure, there was a negligible change in the number of apoptotic cells relative to the untreated control (*P* > 0.99), which increased following 72 h (0.18% more apoptotic cells than the untreated control; 0.44% in total). With respect to the aBL + chloramphenicol combination, at 24 and 72 h after exposure, we found 0.55% and 0.62% apoptotic cells, respectively. This represented a 0.29% and 0.37% more apoptotic cells than the negative control (*P* < 0.001). While the increases in number of in apoptotic cells were statistically significant when treated with aBL alone or aBL + chloramphenicol, the numbers were marginal relative to the population of viable cells, which remained >99% for all groups at all the time points post‐exposure and is above the limit of what might be considered therapeutically safe. We next assessed the impact of aBL alone at terminal radiant exposures that were used during the in vivo efficacy study on the proliferating capability of mouse skin cells. We found that there was a statistically significant effect of aBL on the number of proliferating cells. For example, following both 24 and 72 h, there was a >60% reduction in proliferating cells (*P*<0.001). However, when aBL was combined with chloramphenicol, the impact on proliferating cells was not found to be significant, suggesting that the presence of chloramphenicol limited the side effect of aBL. Additionally, we carried out hematoxylin and eosin staining to determine whether aBL alone or aBL + chloramphenicol altered the skin structure. We did not find any evidence of damage to the skin structure, relative to the untreated control (Figure S5, Supporting Information).

## 3 Discussion

In this study, we assessed, in depth, the potential for aBL to synergize with antibiotics to potentiate their action. In addition, we established, for the first time, the mechanism by which aBL induces bactericidal effects, and detailed the mechanisms by which aBL can promote antibiotic activity. We validated the role of porphyrins in driving aBL activity. Specifically, we found that a Δ*hemB* MRSA USA300 mutant (devoid of porphyrins) was significantly less sensitive to aBL relative to the parental WT strain. Previously, there have been significant studies hypothesizing the contribution of endogenous porphyrins to the bactericidal mechanism in aBL, but until now, this had never been confirmed. For example, in a study by Bumah et al.^[^ ^14^ ^]^ they indirectly found a potential contribution of porphyrins during aBL exposure through use of a porphyrin deficient species and application of exogenous porphyrins and other potential chromophores.

In addition, for the first time we confirmed the direct role of hydroxyl radicals and endogenous iron in promoting bactericidal effects, using *S. aureus* as a model organism. Previous studies have suggested and validated the production of ROS during aBL exposure.^[^ ^7^, ^9^, ^19^, ^20^ ^]^ In our study, however, we delved deeper into the mechanism of ^•^OH radical production and its impact on bacterial viability. We determined that mutant strains that have increased intracellular non‐chelated iron were more susceptible to killing by aBL. We also determined that scavenging ^•^OH radicals or chelating endogenous iron significantly impeded the bactericidal efficacy of aBL. We identified that increases in aBL exposure similarly increased H_2_O_2_ production, thus suggesting that exposure to bacteria to aBL initially induces ROS production (inclusive of H_2_O_2_), which then oxidizes iron via a Fenton reaction to result in ^•^OH production and bacterial death. Previous studies have identified the production of ^•^OH radicals,^[^ ^21^, ^22^ ^]^ however, a detailed look into its contribution to efficacy had not previously been determined.

It is important to appreciate, however, that with aBL it is typical to observe variable efficacies with different bacterial species and even strains within species.^[^ ^23^, ^24^ ^]^ Therefore, it would be important to evaluate the role of porphyrins, ^•^OH production, and “free” iron in other species to identify whether the mechanism of aBL is universal between species. This is particularly true for *P. aeruginosa*, which contains multiple pigments that might potentially be photosensitizing (i.e., pyoverdine that absorbs aBL at 405 nm;^[^ ^25^, ^26^ ^]^ or under certain condition may induce oxidative stress (i.e., pyocyanin).^[^ ^27^ ^]^

As we demonstrated the roles of free iron and ^•^OH production in the bactericidal effects of aBL, we hypothesized that given the proposed similarities to effects caused by antibiotic treatment, there may be potential for synergistic interactions. Initially, we sought to determine whether the rate of killing in bacteria was altered when aBL was combined with antibiotics. For *S. aureus, A. baumannii*, and *E. coli*, we did observe some enhancement in the dose‐response when antibiotics were combined with aBL However, for *P. aeruginosa*, we did not observe any changes to the dose‐response when any of the antibiotics were combined with aBL. It is likely that *P. aeruginosa*, is already highly susceptible to aBL and thus could not be further enhanced by the presence of antibiotics. Potentially, had we increased the concentration of antibiotics beyond 1 x MIC, the dose‐response may have been altered. However, this requires further work to substantiate. Impressively, for all bacteria tested, the inhibitory effects of antibiotics were substantially and synergistically improved by aBL. Additionally, the bactericidal effects of antibiotics were improved post aBL exposure.

We thus hypothesized, that aBL may be priming subsequent inhibition and bactericidal effects of antibiotics. We tested this by exposing bacteria to aBL in the presence of antibiotics and quantifying CFU post‐exposure for a total of 8 h to determine whether both antibiotic inhibition and killing were stimulated by aBL. Indeed, we found that following aBL exposure there was a steady decrease in viability and bacterial growth, confirming that aBL rendered bacteria more susceptible to subsequent antibiotic activity. What is of particular interest is that, even for antibiotics that are bacteriostatic (i.e., chloramphenicol), aBL appeared to promote their bactericidal properties. This observation appears reminiscent of trimethoprim‐sulfamethoxazole which are bacteriostatic antibiotics in isolation. However, when combined, they elicit bactericidal effects, due to inhibiting folic acid biosynthesis at 2 sequential steps of the pathway.

Therefore, given the potential for both antibiotics and aBL to generate ROS, we tested whether increases in ROS may be responsible for this potentiation. We found that, in *A. baumannii* and *S. aureus*, aBL+antibiotics did increase the overall ROS relative to aBL and antibiotics alone. However, it is important to appreciate that only two separate antibiotics were tested in two bacterial species, and thus further work evaluating this effect in other species and strains would be important to fully confirm this hypothesis.

In addition, we hypothesized that increased antibiotic uptake might be responsible for this priming effect that was observed by aBL + antibiotics. Specifically, as we observed that antibiotics that are strictly functional in Gram‐positive antibiotics (i.e., methicillin and vancomycin) appeared to be significantly promoted in Gram negative bacteria such as *P. aeruginosa* and *A. baumannii*. Given that the impermeability of the outer membrane of Gram‐negative bacteria prevents binding of these antibiotics to the cell wall, we accurately predicted that aBL would permeabilize the membrane, a finding that has been validated by other studies.^[^ ^9^, ^19^, ^28^ ^]^ This may be explained by the intrinsitc porphyrins interaction with the bacterial membrane that may potentially induce damage.^[^ ^29^, ^30^ ^]^

Using a fluorescently labeled vancomycin conjugate, we observed that in both *A. baumannii and P. aeruginosa* there was a larger sub‐population of bacteria that accumulated vancomycin when bacteria were pre‐exposed to aBL, suggesting that permeabilization of the membrane facilitated uptake of antibiotics, which explains the observed functionality of these antibiotics in Gram‐negative bacteria. While we observed increases in the bacterial population that accumulated vancomycin, we did not observe, at a single cell level, any significant increases in the uptake of vancomycin. Conversely, in a previous study, we found both *A. baumannii* and *P.aeruginosa* took up more quinine hydrochloride (Q‐HCl) when exposed to aBL relative to Q‐HCl alone.^[^ ^31^ ^]^ This is likely due to the Q‐HCl accumulating within the cytoplasm relative to the peptidoglycan layer as would be observed with vancomycin. In Gram‐negative bacteria there exists a relatively thin peptidoglycan layer that likely becomes rapidly saturated by vancomycin, thus limiting significant increase in vancomycin binding. Further work would be necessary to substantiate this hypothesis.

Unlike with the Gram‐negative bacteria, with *S. aureus* we did not find aBL to increase the population of bacterial cells that accumulated vancomycin. In fact, we found that aBL exposed bacteria reduced the population of bacteria that accumulated vancomycin. However, we did find that, at a single cell level, each individual *S. aureus* cell took double the amount of vancomycin when pre‐exposed to aBL. It is feasible that aBL may have, in some of the population, damaged the motif within the d‐Ala‐d‐Ala dipeptide of the PG‐stem structure that may have limited antibiotic binding, although further work is needed to validate this hypothesis. The increased uptake of vancomycin at a single cell level, may be explained by staphyloxanthin (STX; a carotenoid pigment that anchors to the membrane in *S. aureus*) photolysis^[^ ^21^, ^32^ ^]^ that may permit entry of antimicrobial molecules more effectively.^[^ ^33^ ^]^

Last, we sought to determine the feasibility of using aBL + antibiotics to treat an infection in vivo, using a mouse model of *A. baumannii* burn infection. We initially found that the dose‐response of aBL alone versus aBL + chloramphenicol to be equivalent, as demonstrated by the similar reduction in bioluminescence, which has previously been shown to be correlated with CFU.^[^ ^34^ ^]^ However, when the infected tissues were harvested 24 h post‐treatment, we saw a significant reduction in the viability of *A. baumannii* in mouse burns, relative to aBL or chloramphenicol alone. For the aBL alone group, following 24 h after treatment, there was a regrowth of bacteria. This finding was like what was observed in our in vitro studies. When aBL exposure was discontinued in *A. baumannii*, it was followed by a steady recurrence of viable bacteria during the 8 h experiment, where all bacteria re‐grew. It is likely that during aBL treatment, there was an initial loss of *A. baumannii* viability. However, once aBL exposure was discontinued, the viability steadily increased back to what was observed initially. A likely explanation is that aBL induces ROS (including ^•^OH production) rapidly during exposure, however, once ceased, the bacteria could begin to re‐grow if not eliminated. These findings mirror those presented in photodynamic therapy studies, which have suggested that microbicidal effects only occur during illumination.^[^ ^35^ ^]^ In contrast, in the aBL + chloramphenicol group, it is likely that aBL “primed” the bacteria to become more susceptible to chloramphenicol which in turn permitted the antibiotics to “mop up” remaining bacteria following aBL illumination. It is important to appreciate, however, that different antibiotics may respond differently when exposed to aBL due to variable mechanisms and targets. Therefore, it is critical that these mechanistic processes are evaluated using other antibiotics/bacterial species, in order to determine whether these observations may be globalized.

With respect to safety, we found there to be some increase in caspase‐positive bacteria, relative to the untreated control, when exposed to aBL alone or aBL + chloramphenicol. However, the total percentage loss of viability did not exceed 0.62%, signifying that > 99% of cells remained viable. It has been shown that losses of viability of up to >50% is considered a significant loss of viability,^[^ ^36^ ^]^ suggesting that the marginal loss of viability by aBL alone or aBL + chloramphenicol would likely be acceptable. Our findings mirror numerous studies that have validated the safety of aBL in vitro,^[^ ^37^, ^38^, ^39^ ^]^ in preclinical studies,^[^ ^7^, ^40^ ^]^ and in clinical trials.^[^ ^41^, ^42^ ^]^

Relating to the proliferating capability of mouse skin cells, we did find an effect on the number of proliferating cells when exposed to aBL. In contrast, we found in a previous study conducted in our lab that aBL did not affect cellular proliferation.^[^ ^7^ ^]^ A likely explanation for this difference is that we used a significantly higher irradiance (180 mW cm^−2^ compared with 100 mW cm^−2^), which may potentially be more harmful to cells. Another potential explanation is that chloramphenicol may have attenuated delivery of aBL into the tissue which may have limited its negative effect. Interestingly, we found that when aBL was exposed with chloramphenicol, the impact on cellular proliferation was less, which may have been due to the presence of chloramphenicol attenuating the high irradiance that was used during our experiments.

## 4 Conclusion

In conclusion, our study has shed light on numerous previously unknown processes that occur during aBL treatment and validated the potential of combining aBL and antibiotics for efficient treatment of bacterial infection. In addition, we have illuminated, in detail, the mechanisms involved when combining aBL with antibiotics. Thus, if appropriately optimized, aBL could be a tool within our arsenal to prolong the effectiveness of currently used antibiotics.

## 5 Experimental Section

### Bacterial Species, Strains, and Culture Conditions

A comprehensive list of bacterial species and strains used in the study can be found in Tables S1 and S2 (Supporting Information). In brief, for the initial aBL mechanism studies we used MRSA USA300 and defined mutants generated from this parental wild‐type strain. For aBL/antibiotic combination in vitro studies, we used multidrug‐resistant strains of *S. aureus*, *A. baumannii*, *P. aeruginosa*, and *E. coli*, that were sourced from the CDC isolate bank. For the in vivo studies, we used a bioluminescent variant of *A. baumannii* (ATCC BAA 747 transformed with luxCDABE operon).^[^ ^34^ ^]^ For all experiments, bacteria were prepared initially in brain heart infusion (BHI) medium (broth/agar) and grown in an orbital shaker (100‐120 rpm) or stationary incubator at 37 °C for 24 h.

### Blue Light Sources

For all experiments we used a single light emitting diode (LED) with a peak emission of 405 nm (M405L3; Thorlabs, USA), and a full width at half maximum of 25 nm. The irradiance for all in vitro experiments was set to 60 mW cm^−2^, and the in vivo experiment was set to 180 mW cm^−2^ and the LED was coupled with a collimator (SM2F32‐A; Thorlabs, USA). The irradiance was determined with the use of a PM100D power/energy meter (Thorlabs, USA).

### Blue Light Killing of MRSA Strains and Mutants and Effects of Hydroxyl Radical Scavenging and Iron Chelation In Vitro

Bacteria (see Table S1, Supporting Information) that were grown to stationary phase in BHI (24 h; 10 mL) were diluted in ≈1 mL fresh Luria‐Bertani (LB) broth (Sigma–Aldrich, USA) to an OD_600_ of 0.1. For the hydroxide radical scavenging assay, thiourea (Sigma, USA) was added to the bacterial inoculum at a final concentration of 150 µM. For the iron chelator assay, 2,2′‐Dipyridyl (Sigma, USA) was added to the bacterial inoculum at a final concentration of 120 µM. Samples were transferred to a transparent 48‐well microtiter plate (Costar, USA), and immediately exposed to aBL at radiant exposures of 18, 36, 54, 108, and 216 J cm^−2^, reflecting, 5, 10, 15, 30, and 60 min, irradiation time, respectively. Following each aBL aliquot, 20 µL of bacteria were extracted from the wells and plated for CFU, as described previously.^[^ ^43^ ^]^ Briefly, 10 µL were placed into 90 µL of PBS and serially diluted until a dilution factor of 10^−7^ was achieved. A total volume of 10 µL was then transferred onto the plate and incubated for 18–24 h prior to enumeration.

### Total Intermediate Porphyrins

Using Ultra‐Performance Liquid Chromatography (UPLC), intermediate porphyrins were quantified in USA300 and USA300 *hemB::Tn(ermB)* strains. In summary, overnight cultures (20 mL cultures in 3×50 mL tubes) were collected by centrifugation (10 min at 4,000 rpm), resuspended in 1 mL of extraction buffer (ethanol, dimethyl sulfoxide, acetic acid, 80: 20: 1, vol/vol/vol), and stored at 80 °C for 24 h, followed by disruption process in an ultrasonic bath (M2800; Branson Ultrasonics, USA) for 30 min. Samples were centrifuged and the supernatants were collected for UPLC analysis. The analysis was conducted using a Waters Acquity UPLCTM system as previously described.

### Streptonigrin killing Assay

*Staphylococcus aureus* strains were incubated overnight in tryptic soy broth (TSB) at 37 °C, shaking at 250 rpm. Overnight cultures were adjusted to an OD_600_ of 0.05 using TSB, and 100 µl of adjusted culture were added to 4 mL of 0.3% molten tryptic soy agar (TSA) maintained at 45 °C. The TSA‐culture was mixture and poured on top of a 1.5% TSA plate and allowed to solidify. The center of the soft agar plates was spotted with 2.5 µL of 1 mg mL^‐1^ streptonigrin (Sigma–Aldrich). The soft agar plates were incubated at 37 °C for 20 h. The diameter of the zone of inhibition diameter was determined. The assay was performed with five replicates.

### Blue Light Combined with Antibiotics for Treatment of Bacteria In Vitro

Bacteria (see Table S2, Supporting Information) were grown overnight (stationary phase) prior to diluting in 10 mL LB broth. Immediately, the antibiotics (chloramphenicol, ceftazidime, levofloxacin, methicillin, vancomycin, or sulfamethoxazole) were added at concentrations reflecting the MIC to concentrations 128‐fold below the MIC (Table S3, Supporting Information). In strains where the MIC was above 1024 µg mL^−1^, we considered the maximum concentration (*i.e*., 1024 µg mL^−1^) as 1 x MIC. Immediately following addition of the antibiotic, 1 mL of bacterial suspension was transferred into a 48‐well microtiter plate (Costar, USA) and exposed to aBL at 18, 36, 54, 108, and 216 J cm^−2^, reflecting 5, 10, 15, 30, and 60 min irradiation time, respectively. Following each aliquot of aBL, 20 µL samples were removed and serially diluted prior to plating for CFU.^[^ ^43^ ^]^ Bacterial suspensions samples treated with antibiotic alone, or no antibiotic, or aBL alone were also included as controls and sampled as above. Following treatment, microtiter plates were incubated for 24 h at 37 °C in a stationary incubator. Subsequently, the microtiter plates were removed from the incubator and bacterial growth was assessed using a Spectramax plate reader (M4, Molecular Devices, USA) to measure absorbance at λ = 600 nm. In parallel, the CFU of each of the samples was taken to gauge post‐aBL bactericidal effects.

### Bacterial Bactericidal and Inhibitory Kinetics Following Blue Light and Antibiotics In Vitro

*S. aureus* (AR0215) and *A. baumannii* (AR0083) (see Table S2, Supporting Information) were grown overnight (stationary phase) prior to diluting in 10 mL LB broth. For the bactericidal kinetics studies using *S. aureus*, the LB broth was supplemented with either chloramphenicol or ceftazidime at 1 x MIC (Table S3, Supporting Information). For *A. baumannii* bactericidal studies, the LB broth was supplemented with either chloramphenicol or vancomycin. Bacterial suspensions were initially plated for CFU as above, and subsequently exposed to either 18 J cm^−2^ aBL (*A. baumannii*) or 108 J cm^−2^ (*S. aureus*), prior to incubating at 37 °C. Bacteria were then quantified every hour for a total of 8 h in order to gauge viability over time. For the inhibition studies, bacteria were exposed to the same antibiotics as described above. Bacteria were exposed to either 18, 36, 54, 108, or 216 J cm^−2^, reflecting 5, 10, 15, 30, or 60 min of aBL, prior to transferring 200 µL of the bacteria/LB/antibiotic mix to a 96‐well microtiter plate (Falcon, USA) and measuring the OD_600_ every 30 min for a total of 14 h.

### Quantification of Hydrogen Peroxide, Hydroxyl Radicals, and Generic Reactive Oxygen Species Production

Hydrogen peroxide was assessed in MRSA USA300 and its defined Δ*hemB* mutant using the 10‐acetyl‐3,7‐dihydroxyphenoxazine assay (Amplex Red; Invitrogen, USA) in accordance with manufacturer's instructions. In brief, bacteria were exposed to increasing aBL doses, including: 0, 18, 36, 54, 108, and 216 J cm^−2^, reflecting to: 0, 5, 10, 15, 30, and 60 min. After light exposure, 50 µL of Amplex red reagent in working solution were add to each well in 96‐well, black, optically clear flat‐bottom plate (Nunc, Thermo Scientific, USA) containing the 50 µL of samples. The plate was incubated at room temperature for 30 min in the dark, followed by fluorescence measurements (λ_ex_ = 530 and λ_em_ = 560 nm) using the Spectramax plate reader (M4, Molecular Devices, USA).

Generic ROS production was quantified in *S. aureus* (AR0215) and *A. baumannii* (AR0083) using 2′, 7′‐dichlorofluorescein diacetate (DCFH‐DA; D6883, Sigma–Aldrich) as per the instructions. In brief, 2 µM of DCFH‐DA was added to one mL of bacterial inoculum, in the presence or absence of levofloxacin or vancomycin. Bacteria were then exposed to increasing aBL exposures including: 0.9, 1.8, 3.6, 7.2, 18, 36, and 54 J cm^−2,^ which reflected, 0.25, 0.5, 1, 2, 5, 10, and 15 min, respectively. Following each light exposure aliquot, fluorescence was determined (λ_ex_ = 504 and λ_ex_ = 529 nm) using the Spectramax plate reader (M4, Molecular Devices, USA).

### Bacterial Permeabilization Studies

For the outer membrane permeabilization assay, suspensions of *A. baumannii* (AR0083), *E. coli* (AR0541), and *P. aeruginosa* (AR0246) were adjusted to an OD_600_ of 0.1 into Luria‐Bertani (LB) medium. Samples were transferred to a 48‐well microtiter plate (Costar, USA) and exposed to aBL at radiant exposures of 18, 36, 54, 108, and 216 J cm^−2^, reflecting 5, 10, 15, 30, and 60 min of irradiation time. Positive control (Polymyxin B at 10 ug mL^−1^ for 30 min) and negative control (untreated sample) were included for each strain. The bacterial samples were harvested by centrifugation (10 min at 4,000 rpm) and resuspended in PBS. N‐phenyl‐1‐naphthylamine (NPN; Sigma Aldrich, USA) was added to each sample at a final concentration of 15 µM and incubated in dark conditions at room temperature for 15 min. Samples were transferred into a 96‐well, black, optically clear flat‐bottom plate (Nunc, Thermo Scientific, USA), and the NPN bacterial incorporation was measured by fluorescence (at λ_ex_ = 350 nm and λ_em_ 415 nm) using a plate reader spectrophotometer (SpectraMax M4; Molecular Devices, USA).

### Vancomycin Accumulation Studies

In this study, we used *S. aureus* (AR0215), *A. baumannii* (AR0083), or *P. aeruginosa* (AR0246). Bacteria were grown to stationary phase, prior to diluting bacteria to a 0.5 OD_600_ in LB broth and transferring 1 mL of bacterial suspensions to a 48‐well microtiter plate (Costar, USA). For *A. baumannii* and *P. aeruginosa*, bacteria were exposed to 54 J cm^−2^ aBL (15 min) prior to applying 2 µg mL^−1^ of a fluorescent vancomycin‐bodipy conjugate (BODIPY™ FL Vancomycin, Invitrogen™, USA). For *S. aureus*, 108 J cm^−2^ was pre‐exposed prior to application with the fluorescent vancomycin‐bodipy conjugate. Bacteria were then incubated for 1 h at 37 °C in a stationary incubator. Subsequently, bacteria were centrifuged at 4000 x *g* for 5 min prior to resuspending the pelleted bacteria in 1 mL PBS. This was repeated a further 2 times, in order to wash the bacteria of the exogenous vancomycin‐bodipy conjugate. Then, bacteria were resuspended in 200 µL PBS, prior to further diluting 1000‐fold in PBS at a final volume of 200 µL. The suspension (100 µL) was then transferred to a glass bottom 96‐well plate (Cellvis, USA). The bacterial vancomycin‐BODIPY uptake was analyzed by fluorescence microscopy using a green fluorescent protein filter (Olympus FV1000‐MPE Confocal, USA).^[^ ^44^ ^]^ Relative fluorescence and particle analyses was determined using image processing software (ImageJ 1.53, National Institutes of Health, USA).

### Combination of Blue Light and Chloramphenicol Against *A. Baumannii* Burn Infection In Vivo

Female BALB/c mice aged 6–8 weeks were used. Initially, mice were anaesthetized with a ketamine/xylazine cocktail (100‐20 mg k^−1^g; i.p), and subsequently shaved. Mice were given analgesics in the form of buprenorphine (0.1 mg k^−1^g; IP) prior to injury. A 3^rd^ degree burn was then established via the vertical application of a 1.2×1.2 cm brass block that was heated in water to >95 °C on the dorsal surface of each mouse. This application lasted 3 s. Subsequently, the burned skin was left to cool for 5 min, prior to application of approximately 10^8^ CFU of *A. baumannii* ATCC BAA 747 which was transformed with *luxCDABE* operon. Mice were kept separated until the bacterial inoculum dried, and then mice were returned to their housing for another 24 h. Subsequently, mice were anaesthetized with the ketamine/xylazine cocktail, prior to applying, topically, a 0.1% chloramphenicol solution (200 µL), which was left to permeate the wound for 30 min. Subsequently, mice were exposed to different aBL aliquots, including, 54, 108, 162, 216, 270, and 324 J cm^−2^, reflecting, 5, 10, 15, 20, 25, and 30 min of exposure, respectively. Following application of aBL, a further 200 µL of the 0.1% chloramphenicol solution was applied. Mice treated with chloramphenicol alone, aBL alone, or vehicle control (PBS) were also included in the same manner as described above. Mice were then returned to their housing for a further 24 h, prior to euthanizing the mice, and isolating the infected skin tissue for subsequent homogenization, using Lysing Matrix D tubes (MP Biomedicals, USA) within a homogenizer (FastPrep‐24™ Biomedicals, USA) and CFU determination.^[^ ^7^ ^]^

### Histological and Immunohistochemical Studies

The cell proliferation and apoptosis activity following blue light combined with chloramphenicol were assessed in naïve mice skin. In brief, 0.1% chloramphenicol solution (200 µL) was applied to the skin 30 min previously to the light exposure to allow complete penetration. The animals were then exposed to 324 J cm^−2^, reflecting 30 min of aBL. Following the light exposure, 200 µL of the 0.1% chloramphenicol solution was re‐applied. For comparison treatments, mice applied with chloramphenicol alone, aBL alone, or vehicle control (PBS) were also included.

The animals were euthanized 24 or 72 h after treatment, and the skin was collected and fixed in 10% phosphate‐buffered formalin (Fisher Scientific, USA) prior to immunohistochemical processing. A Ki‐67 antibody (ab 16 667; Abcam, USA) conjugated to a probe labelled with fluorescent Cy3 (λ_ex_ = 578 and λ_em_ 603 nm) was used for the cell proliferation assay. The caspase‐3 antibody (966; Cell Signaling, USA) was used to evaluate apoptosis conjugated to a fluorescent Cy5 labeled probe (λ_ex_ = 658 and λ_em_ 675 nm) was used to evaluate apoptosis. The immunofluorescence sections were imaged by Nanozoomer S60 digital slide scanner (C13210‐ 01; Hamamatsu, USA). The percentage fluorescence of Ki‐67 (at 578 nm) or caspase‐3 (at 675 nm) relative to the entire analyzed area was analyzed using ImageJ (ImageJ 1.53, National Institutes of Health, USA) according to the previously described.^[^ ^7^ ^]^ In addition, hematoxylin and eosin (H&E) staining performed to assess cellular or structural changes in the skin.

### Animal Experiments

All animal procedures were approved by the Institutional Animal Care and Use Committees of Massachusetts General Hospital (protocol number: 2015N000187) in accordance with National Institute of Health guidelines.

### Statistical Analyses

Data were presented as the mean ± the standard error of the mean (SEM). All CFU data were normalized into their log_10_ values prior to their analyses. Statistical tests included a paired t‐test, one‐way, or two‐way *analysis of variance* (ANOVA) with post‐hoc Bonferroni corrections comparing either (aBL vs aBL + antibiotics or antibiotics vs aBL + antibiotics). All analyses were performed using GraphPad Prism 9.0.

## Conflict of Interest

The authors declare no conflict of interest.

## Author Contributions

L.G.L. and C.D.A contributed equally to this work. L.G.L., R.R.A., and T.D. were responsible for study design and conception. L.G.L., C.D.A., J.M.B., and T.D. developed the methodology. L.G.L., C.D.A, K.R.K., and J.H. acquired the data, while L.G.L., C.D.A., J.M.B., and T.D. were responsible for interpretation of data. L.G.L, C.D.A., and T.D. were responsible for writing the original draft and. L.G.L., J.M.B., R.R.A., D.C.H. and T.D. were responsible for reviewing and editing.

## Supporting information

Supporting Information

## Data Availability

The data that support the findings of this study are available from the corresponding author upon reasonable request.
