# Supplementary material for: Blue Light Potentiates Antibiotics in Bacteria via Parallel Pathways of Hydroxyl Radical Production and Enhanced Antibiotic Uptake
Source: Adv Sci (Weinh). 2023 Nov 9;10(36):2303731. doi: 10.1002/advs.202303731 (PMC10754126; doi:10.1002/advs.202303731)
Supplement: Supplementary file 1 — Supporting Information [file ADVS-10-2303731-s001.pdf]

ADVANCED SCIENCE
Open Access

Supporting Information

for *Adv. Sci.*, DOI 10.1002/advs.202303731

Blue Light Potentiates Antibiotics in Bacteria via Parallel Pathways of Hydroxyl Radical Production and Enhanced Antibiotic Uptake

*Leon G. Leanse, Carolina dos Anjos, Kylie Ryan Kaler, Jie Hui, Jeffrey M. Boyd, David C. Hooper, R. Rox Anderson and Tianhong Dai**

Supplementary Materials for

Blue light potentiates antibiotics in bacteria via parallel pathways of hydroxyl radical production and enhanced antibiotic uptake

Leon G. Leanse *et al.*

*Corresponding author. Email: tdai@mgh.harvard.edu

This PDF file includes:

Figs. S1 to S5
Tables S1 to S3

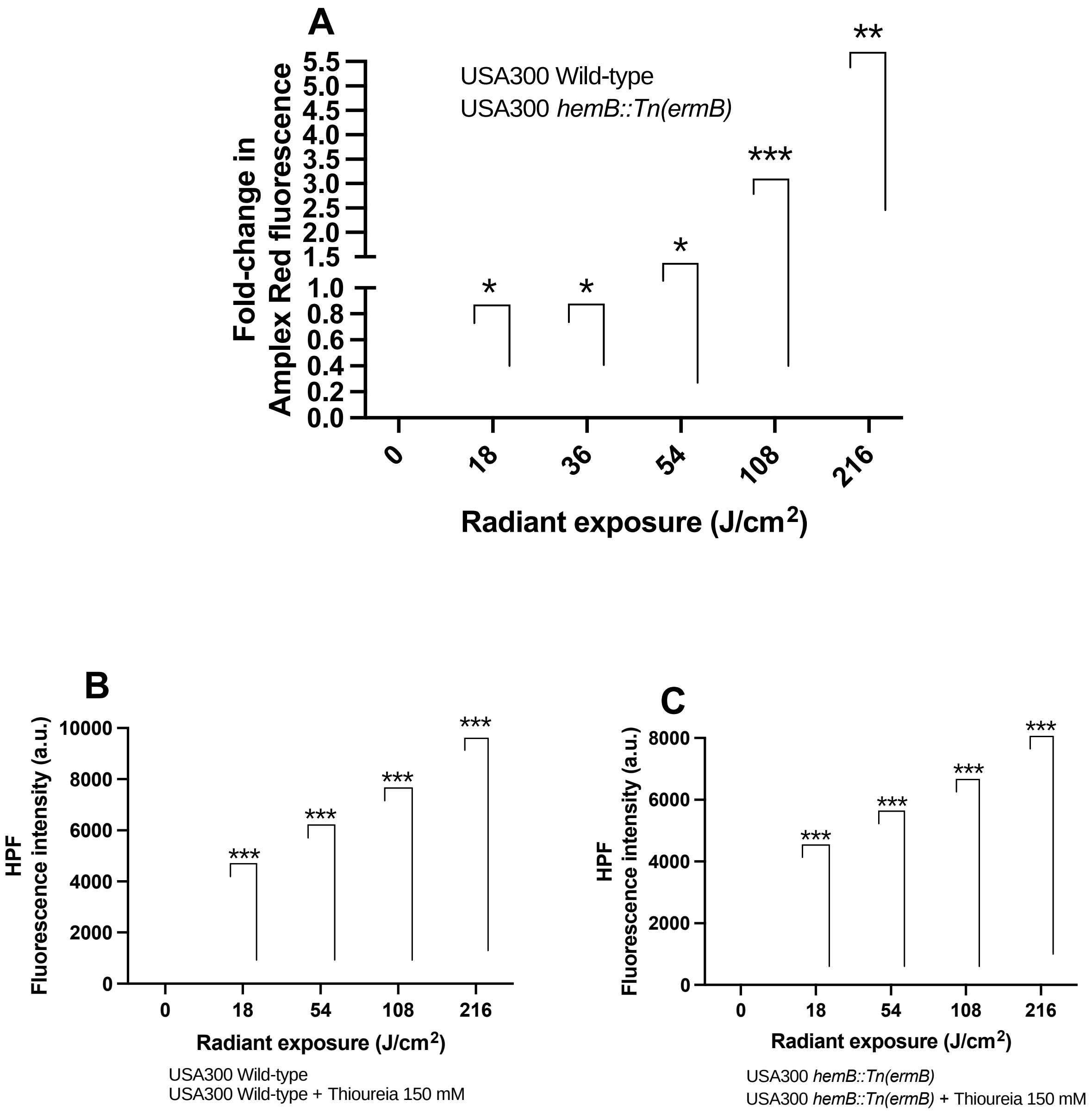

Fig. S1. Detection of hydrogen Peroxide and hydroxide radical comparing USA 300 Wild-type and USA300 hemB::Tn after increasing antimicrobial blue light. Detection of (A) H_2O_2 using the Amplex® Red Hydrogen Peroxide assay comparing USA 300 Wild-type and USA300 *hemB::Tn* after increasing antimicrobial blue light (aBL) exposure. Fluorescence was measured with a fluorescence microplate reader using excitation 350 nn and emission detection at 590 nm. Detection of •OH radicals following aBL (with or without thiourea) in the USA 300 Wild-type (B) and (C) USA300 *hemB::Tn* with the use of the using hydroxyphenyl fluorescein (HPF) sensor. Fluorescence was measured with a fluorescence microplate reader using an excitation/emission: 490/515 nm. For the H_2O_2 studies Data are presented as means ± SEM of fold-change in Amplex Red fluorescence, and for the HPF studies the fluorescence intensity changes were evaluated. *$p<0.05$, **$p<0.01$, ***$p<0.001$.

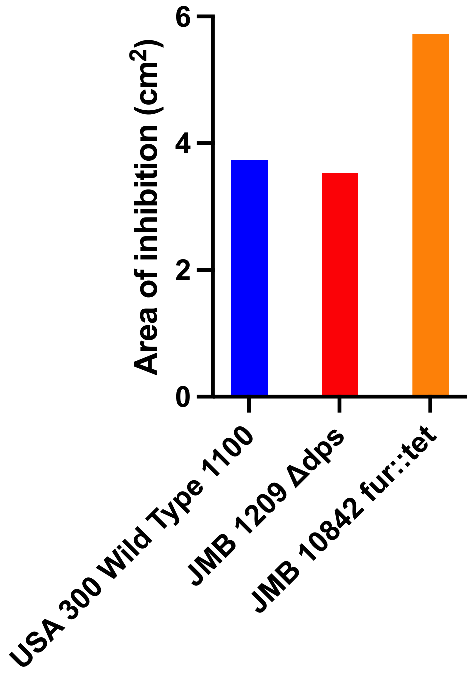

Fig. S2. Bacterial growth inhibition following streptonigrin treatment. Quantification of the area of growth inhibition of bacterial strains following treatment with streptonigrin.

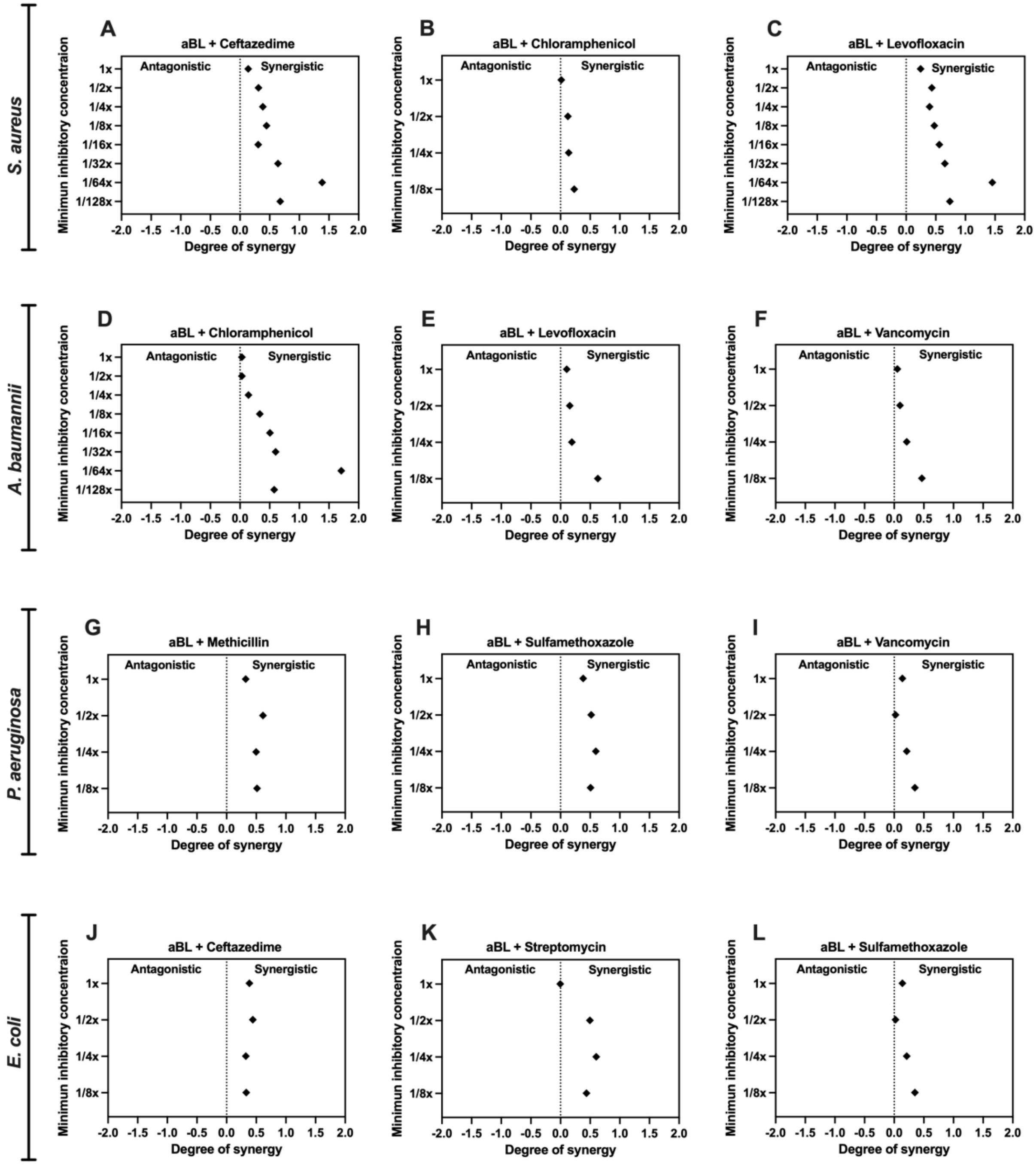

Fig. S3. Synergism of aBL and antibiotic combination. Value > 0 means synergistic combination, value < 0 means antagonism combination. Type or paste caption here. Create a page break and paste in the Figure above the caption.

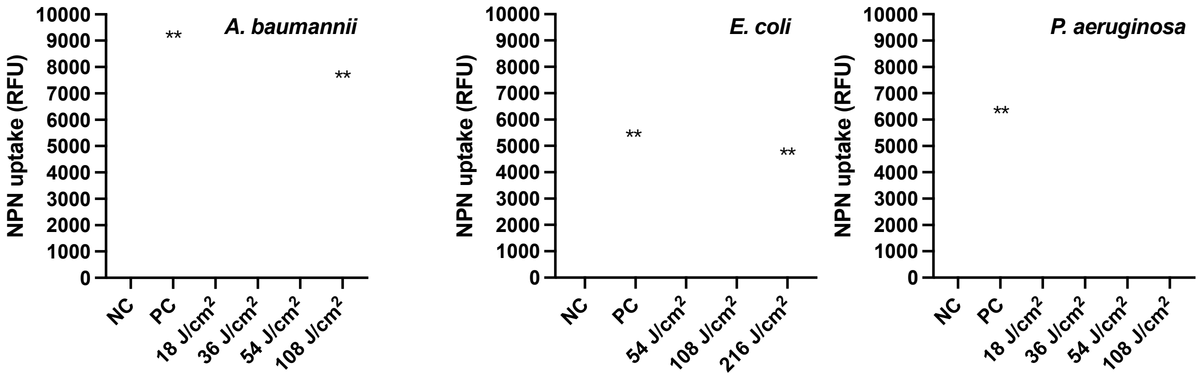

Fig. S4. Effects of aBL on membrane permeabilization in *A. baumannii, E. coli* and *P. aeruginosa* assessed by N-phenyl-1-naphthylamine (NPN). Values are presented as means ± SEM. *p<0.05, **p<0.01, ***p<0.001 compared to the negative control (NC). PC is positive control.

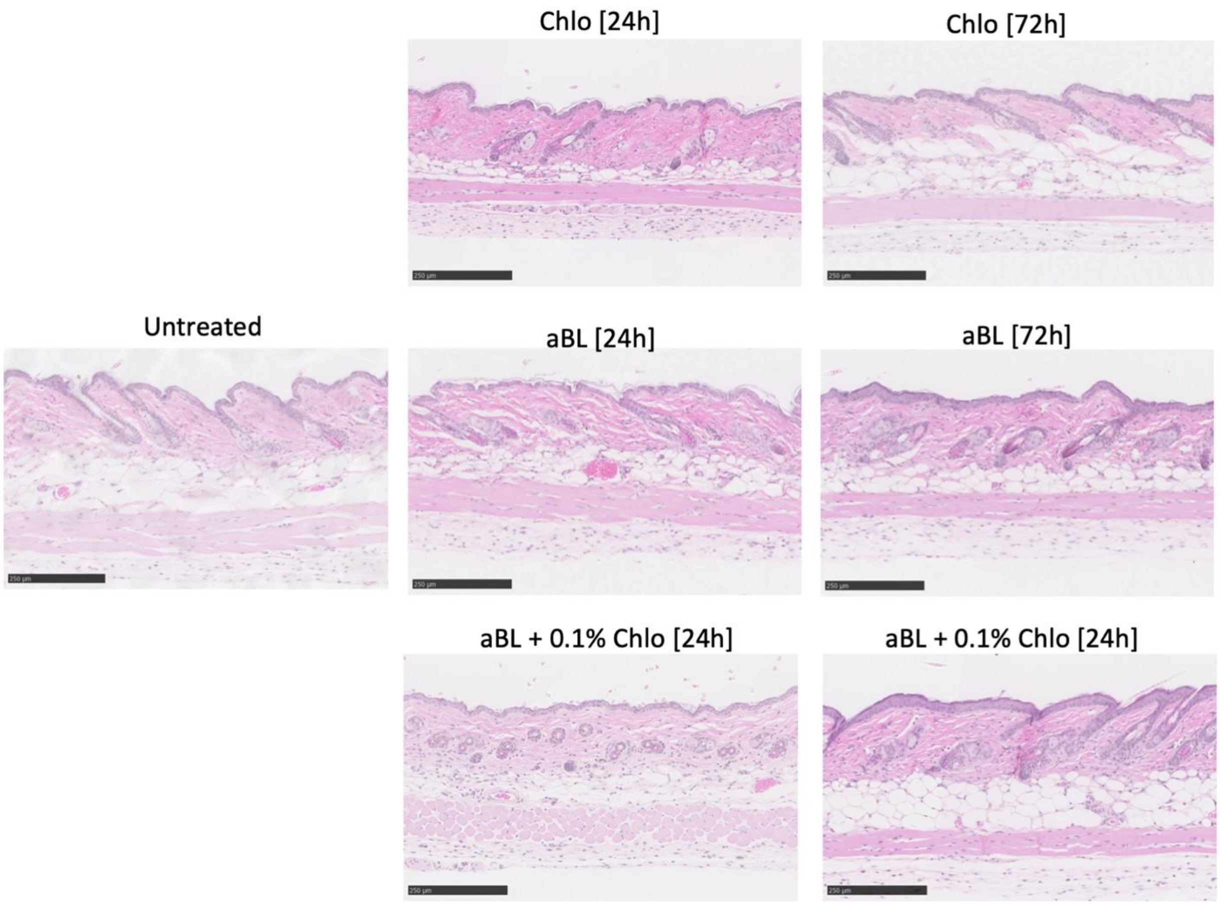

Fig. S5. H**ematoxylin and eosin-stained histological sections of mouse naïve skin exposed to antimicrobial blue light (324 J/cm^2).** Skin samples were collected after 24 and 72h of light exposure. Bar: 250 µm.

***S. aureus* Strains**	**Genotype description**	**Source/Reference**
JMB1100	Boyd lab USA300_LAC wild-type	Mashruwala *et al*., 2015; PMID: 25388433
JMB6037	*hemB::Tn(ermB)*	Mashruwala *et al*., 2017; PMID: 28221135
JMB10842	*fur::tetM*	Mashruwala *et al*., 2016; PMID: 27671355
JMB8464	*sufD**	Roberts *et al.*, 2017; PMID: 28320837
JMB8472	*sufD** pLL39_*suf*	Roberts *et al.*, 2017; PMID: 28320837
JMB1209	Δ*dps*	Mashruwala *et al*., 2017; PMID: 28099473
JMB11633	*addB::Tn(ermB)*	Roberts *et al.*, 2017; PMID: 28320837
JMB11634	*addB::Tn(ermB) Δdps*	(This work)

Table S1. Strains used in this study. USA300 and defined mutants generated from this parental wild-type strain.

AR Bank #	Specie	Resistance category	Resistance gene	Antimicrobial susceptibility
AR0215	*Staphylococcus aureus*	Aminoglycoside<br>Beta-lactam<br>Macrolide-Lincosamide-Streptogramin	aadD,spc<br>blaZ,mecA<br>erm(A)	Ceftaroline, Gentamicin, Linezolid, Rifampin, Tetracycline, Trimethoprim/sulfamethoxazole
AR0083	*Acinetobacter baumannii*	Aminoglycoside<br>Beta-lactam<br>Macrolide-Lincosamide-Streptogramin<br>Phenicols/Bicyclomycins<br>Rifampicin<br>Sulfonamides<br>Tetracyclines<br>Trimethoprim	aph(3')-Ic,armA,strA<br>NDM-1,OXA-23,OXA-69,PER-7<br>mph(E),msr(E)<br>cmlA1<br>ARR-3<br>sul1,sul2<br>tet(B)<br>dfrA1	Colistin
AR0246	*Pseudomonas aeruginosa*	Aminoglycoside<br>Beta-lactam<br>Tetracyclines	aadB,rmtD2<br>NDM-1,OXA-10,OXA-50,PAO,VEB-1<br>tet(G)	Colistin
AR0541	*Escherichia coli*	Aminoglycoside<br>Beta-lactam<br>Macrolide-Lincosamide-Streptogramin<br>Sulfonamides<br>Tetracyclines<br>Trimethoprim	aadA5<br>KPC-3,CTX-M-15,OXA-1,TEM-1B<br>mph(A)<br>sul1<br>tet(A)<br>dfrA17	Cefoxitin, Ceftazidime/avibactam, Doripenem, Eravacycline, Gentamicin, Meropenem, Tigecycline

Table S2. Antimicrobial resistance strains. List of antimicrobial resistance strains acquired from CDC & FDA Antibiotic Resistance (AR) Isolate Bank selected for this study.

**Antibiotic**	***S. aureus*** **AR0215**	***A. baumannii*** **AR0083**	***P. aeruginosa*** **AR0246**	***E. coli*** **AR0541**	***S. aureus WT and mutans***
Ceftazidime	256	>1024	>1024	512	128
Chloramphenicol	32	256	>1024	32	-
Levofloxacin	1024	4	64	32	-
Methicillin	128	512	512	1024	-
Streptomycin	64	>1024	>1024	128	-
Sulfamethoxazole	>1024	>1024	>1024	>1024	-
Vancomycin	1	128	>1024	256	-

Table S3. Minimum inhibitory concentration. The minimum inhibitory concentration (μg/mL) of antibiotics tested in Luria-Bertani broth medium.
